# Portable nanopore-sequencing technology: Trends in development and applications

**DOI:** 10.3389/fmicb.2023.1043967

**Published:** 2023-02-01

**Authors:** Pin Chen, Zepeng Sun, Jiawei Wang, Xinlong Liu, Yun Bai, Jiang Chen, Anna Liu, Feng Qiao, Yang Chen, Chenyan Yuan, Jingjie Sha, Jinghui Zhang, Li-Qun Xu, Jian Li

**Affiliations:** ^1^Key Laboratory of DGHD, MOE, School of Life Science and Technology, Southeast University, Nanjing, China; ^2^China Mobile (Chengdu) Industrial Research Institute, Chengdu, China; ^3^School of Computer Science and Technology, Southeast University, Nanjing, China; ^4^Clinical Laboratory, Southeast University Zhongda Hospital, Nanjing, China; ^5^School of Mechanical Engineering, Southeast University, Nanjing, China

**Keywords:** portable nanopore-sequencing technology, protein nanopore, solid-state nanopore, development, application, challenge

## Abstract

Sequencing technology is the most commonly used technology in molecular biology research and an essential pillar for the development and applications of molecular biology. Since 1977, when the first generation of sequencing technology opened the door to interpreting the genetic code, sequencing technology has been developing for three generations. It has applications in all aspects of life and scientific research, such as disease diagnosis, drug target discovery, pathological research, species protection, and SARS-CoV-2 detection. However, the first- and second-generation sequencing technology relied on fluorescence detection systems and DNA polymerization enzyme systems, which increased the cost of sequencing technology and limited its scope of applications. The third-generation sequencing technology performs PCR-free and single-molecule sequencing, but it still depends on the fluorescence detection device. To break through these limitations, researchers have made arduous efforts to develop a new advanced portable sequencing technology represented by nanopore sequencing. Nanopore technology has the advantages of small size and convenient portability, independent of biochemical reagents, and direct reading using physical methods. This paper reviews the research and development process of nanopore sequencing technology (NST) from the laboratory to commercially viable tools; discusses the main types of nanopore sequencing technologies and their various applications in solving a wide range of real-world problems. In addition, the paper collates the analysis tools necessary for performing different processing tasks in nanopore sequencing. Finally, we highlight the challenges of NST and its future research and application directions.

## 1. Introduction

In (1953), Watson and Crick creatively proposed the double helix structure of DNA, as discussed in Watson and Crick (1953), which contributed to the conceptual framework of DNA replication and nucleic acid encoding proteins. This pioneering work ushered in the era of computational molecular biology from descriptive biology (Heather and Chain, 2016), and sequencing technology is the most commonly used technology in molecular biology research and an essential pillar for the development of molecular biology.

In scientific research, sequencing technology plays a significant role in genomics, metagenomics, DNA-protein interaction, and DNA methylation research (Pareek et al., 2011). Sequencing technology has also found wide applications in all aspects of human life. It plays an irreplaceable role in disease prediction, pathological research, drug development, organ transplant matching, prenatal testing, molecular tumor diagnosis, targeted therapy, COVID-19 detection, and more. Since the emergence of sequencing technology, nucleotide detection technology has made considerable progress. In 1977, the first-generation sequencing technology represented by the Maxam-Gilbert method (Liu et al., 2013) and the chain termination method (Garaj et al., 2010) came into being, which opened the door for scientists to explain the genetic code of life. Reads produced by this technology are 700–900 bp in length, and the accuracy rate can reach 99.999% (Menestrina et al., 2014). The first-generation sequencing technology, featuring short running time and high accuracy, is suitable for small-scale scientific research with low throughput requirements, but each reaction can only obtain one read length, which is not suitable for large-scale high-throughput sequencing. In 2005, the second-generation sequencing methods represented by sequencing by synthesis (SBS) (Maxam and Gilbert, 1977) and sequencing by ligation (SBL) (Sanger et al., 1977) began to enter the sequencing market. The typical read length is about 50–500 bp, and the throughput is thousands of times higher than that of the first generation. As a result, the cost of a single sequencing is slashed, opening the door for large-scale sequencing (Suárez, 2017). In 2008, the third-generation sequencing technology, featuring single-molecule sequencing and long-read length, emerged and caught the intense attention of the scientific community. The third-generation sequencing technology applies sequencing-by-synthesis methods to an array of single DNA molecules, avoiding a series of errors caused by PCR amplification. The third-generation sequencing read length can reach 2 Mb, with higher data throughput and lower cost of use (van Dijk et al., 2018), which enables researchers to investigate genomic regions with complex structures, repeat sequences with high or low GC contents, as well as go through small genomes such as virus genome without assembly.

**Figure 1:**
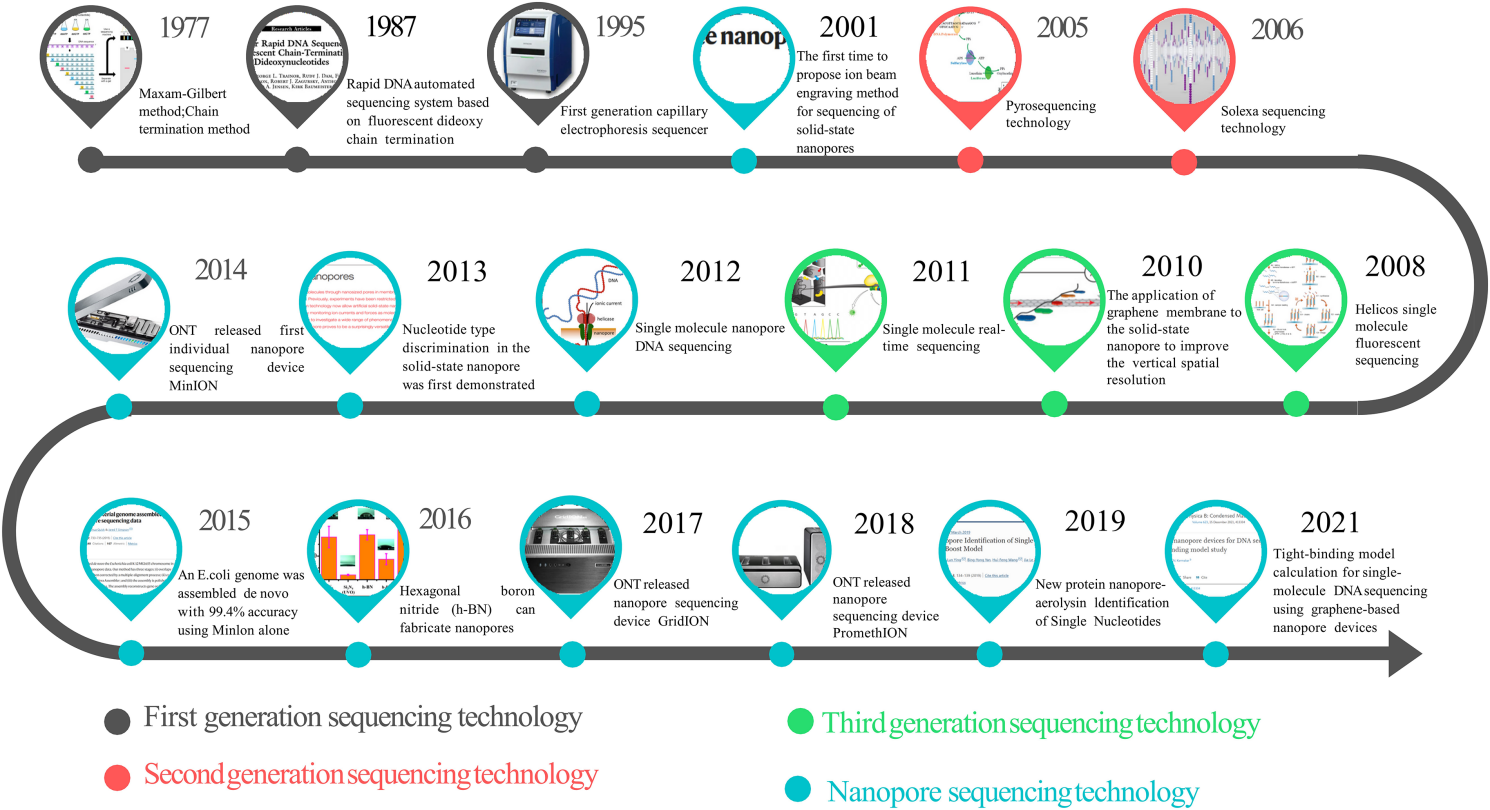
The timeline of sequencing technology development.

Furthermore, because the signal changes caused by the nucleotides after methylation and other modifications are different, third-generation sequencing can detect the methylation modification of genes by detecting electrical signal changes (Petersen et al., 2019), which expands the application scope of sequencing technology. However, because it does not participate in the background interference of free fluorescent factors in the actual chemical reaction, the accuracy of the third-generation sequencing is significantly lower than that of the first and second-generation ones (Schadt et al., 2010). All the generations of sequencing technologies discussed insofar use fluorescent color-developing substances to detect DNA sequences. In the process of DNA polymerase assembling different dNTPs into the DNA chain, different light signals thus generated are detected to determine the base composition of the DNA chain indirectly. The fluorescence detection system required by these methods and the enzyme required for DNA synthesis increase the cost of DNA sequencing (Ambardar et al., 2016). Therefore, the development of new-generation sequencing technology is expected to be small in physical size, not rely on biochemical reagents, and be directly readable by physical methods. And NST is a distinguished representative among them. Figure 1 summarizes the key time points in the development of sequencing technologies across generations. According to MarketsandMarkets Strategic Insights (Next Generation Sequencing Market, 2021), the next-generation sequencing market has been growing rapidly; in 2021, the market size is 10.3 billion US dollars, and by 2026, the next-generation sequencing market is expected to reach 24.2 billion US dollars, with a compound annual growth rate (CAGR) of 18.7% over the years (Next Generation Sequencing Market, 2021).

## 2. The history of biological nanopore sequencing

In a patent authored by Church and Deamer et al. and granted in 1998, they proposed using nanopore sensors to sequence DNA (Church et al., 1998a). Previously, in 1994, Walker et al. found that α-hemolysin is a good metal ion sensor material (Walker et al., 1994). Therefore, α-hemolysin was included as a candidate for nanopore sequencing materials. Bezrukov and Kaasianowicz et al. have accumulated rich experience in applying α-hemolysin. They have mastered the conditions necessary for nanopore sequencing to avoid spontaneous gating (pore closure) of the α-hemolysin channel (Bezrukov and Kasianowicz, 1993). After sufficient technical and theoretical preparations, they set out to conduct initial nanopore sequencing experiments, and the results demonstrated that a single RNA molecule could pass through the α-hemolysin channel (Akeson et al., 1999). In 1994, the X-ray diffraction crystal structure experiments on α-hemolysin further revealed the structure, and the diameter of the α-hemolysin pores is about 2 nm. Therefore, scientists inferred that the DNA that can pass through an aperture of this size was single-strand DNA(ssDNA)instead of double-strand DNA(dsDNA) (Song et al., 1996). Then, researchers at Harvard University tested a mixture of dsDNA and ssDNA and quantified the reaction through the hole by quantitative PCR technology, which was indeed ssDNA instead of dsDNA (Wang et al., 2021).

Furthermore, the study found an exciting result that the number of ionic currents blocked was consistent with the number of translocated ssDNA molecules, suggesting that the use of α-hemolysin for nanopore sequencing is highly possible. In 1996, researchers published the results of the first nanopore sequencing study (Kasianowicz et al., 1996), providing an initial verification of the original idea. At the same time, other papers also investigated if nanopore technology would be capable of detecting low-molecular-weight compounds such as DNA (Cornell et al., 1997).

The next unavoidable problem of α-hemolysin nanopore sequencing is identifying purine bases from pyrimidine bases. In 1997, experiments showed that α-hemolysin nanopores could provide information about oligomeric structure or composition (Akeson et al., 1999). Further experiments revealed that the nanopore signal reaction could distinguish several ssDNA polynucleotides of the same length that differed only in oligomerization sequence and had considerable sensitivity (Meller et al., 2000). Later experiments also confirmed that the pore signal could distinguish if the polynucleotide chain passing through the nanopore was from 5′ to 3′ or from 3′ to 5′ (Wang et al., 2004; Mathé et al., 2005). Finally, in 2005, Ashkenasy et al. found that single-stranded DNA can form a single species of α-hemolysin DNA pseudorotaxane with α-hemolysin protein and, in this process, recognize adenine through changes in α-hemolysin ionic current nucleotides (Ashkenasy et al., 2005). This experiment further demonstrated that α-hemolysin is sufficiently discriminative for all four DNA nucleotide bases, regardless of whether they are located in homopolymeric DNA strands (Stoddart et al., 2009). These experiments have achieved positive results but still have their limitations. The DNA single-strands in the experiments are all stably immobilized near the nanoporin, and it is still questionable whether the detection of free DNA single-strands has sufficient sensitivity.

Early experimental results showed that a single strand of DNA traveled through the nanopore at a rate of 1–10 bases per millisecond, which was way too fast for the detection signal (Akeson et al., 1999). For this reason, scientists hoped to improve the accuracy of reading by controlling the rate, at which ssDNA molecules pass through the nanopore while taking measures in three directions:

1. Controlling the catalytic efficiency of enzymes. In 1998, it was proposed to tune the rate of DNA translocation in nanopores by controlling the catalytic efficiency of enzymes (Church et al., 1998b) and tried a variety of polymerases (Gyarfas et al., 2009); eventually, phi29 DNA polymerase (DNAP) was selected due to its excellent continuous synthesis ability and high affinity for DNA substrates (Lieberman et al., 2010);
2. Modifying the protein nanopore to meet the needs of sequencing. The original α-hemolysin is not the most suitable protein pore object (Sugawara et al., 2015), and many efforts have been made to improve the structural properties of protein nanopores. The nanopore length of the sequencing site of α-hemolysin is about 5 nanometers (nm). Studies have shown that polymers with a longer than nanopore pore size can maintain a uniform translocation rate, while the rate of translocation of polymers with a shorter than nanopore pore size through the nanopore increases with decreasing length (Meller et al., 2001). Therefore, a shorter hole may provide better base discrimination (Ayub et al., 2015). It was found that Mycobacterium smegmatis Porin A (MspA) has similar characteristics to the mechanism of α-hemolysin, which was later proved to have smaller pore width and a more stable pore rate, making it an ideal nanopore sequencing material (Stahl et al., 2001). Further studies also found that MspA can bind metal ions to stabilize protein structure better than α-hemolysin and is an excellent engineering template for detecting extremely small analytes (Wang et al., 2019). Moreover, the MspA nanopore can distinguish all four nucleobases and achieve a significantly more difference in ionic current between nucleobases than α-hemolysin (Laszlo et al., 2016). Further research has developed new insights into making a variety of MspA mutants that can rapidly and inexpensively produce MspA protein nanopores, and the performance of nanopores prepared by this method is not different from that of nanopores prepared by other methods (Yan S. et al., 2021);
3. Changing the physical and chemical properties of the sequencing solution. The ionic conductance of DNA through α-hemolysin and the rate of entry of polynucleotide chains into the nanopore channel decreased with the increase in the viscosity of the sequencing solution (Kawano et al., 2009). Therefore, the speed of DNA strands passing through α-hemolysin can be controlled by adjusting the viscosity of the sequencing solution. At the same time, the study also found that reducing the temperature of the sequencing solution can also reduce the speed of DNA strand translocation through pores, which can be used in combination with various methods, thereby improving the accuracy of sequencing signal reads (Luan et al., 2012). Reasonable monitoring methods are indispensable to achieve precise DNA strand translocation control. A microarray system has been developed to simultaneously monitor multiple ion fluxes of α-hemolysin nanopores during high-throughput sequencing (Osaki et al., 2009).

## 3. Nanopore sequencing technology from the laboratory to practical application

**Figure 2:**
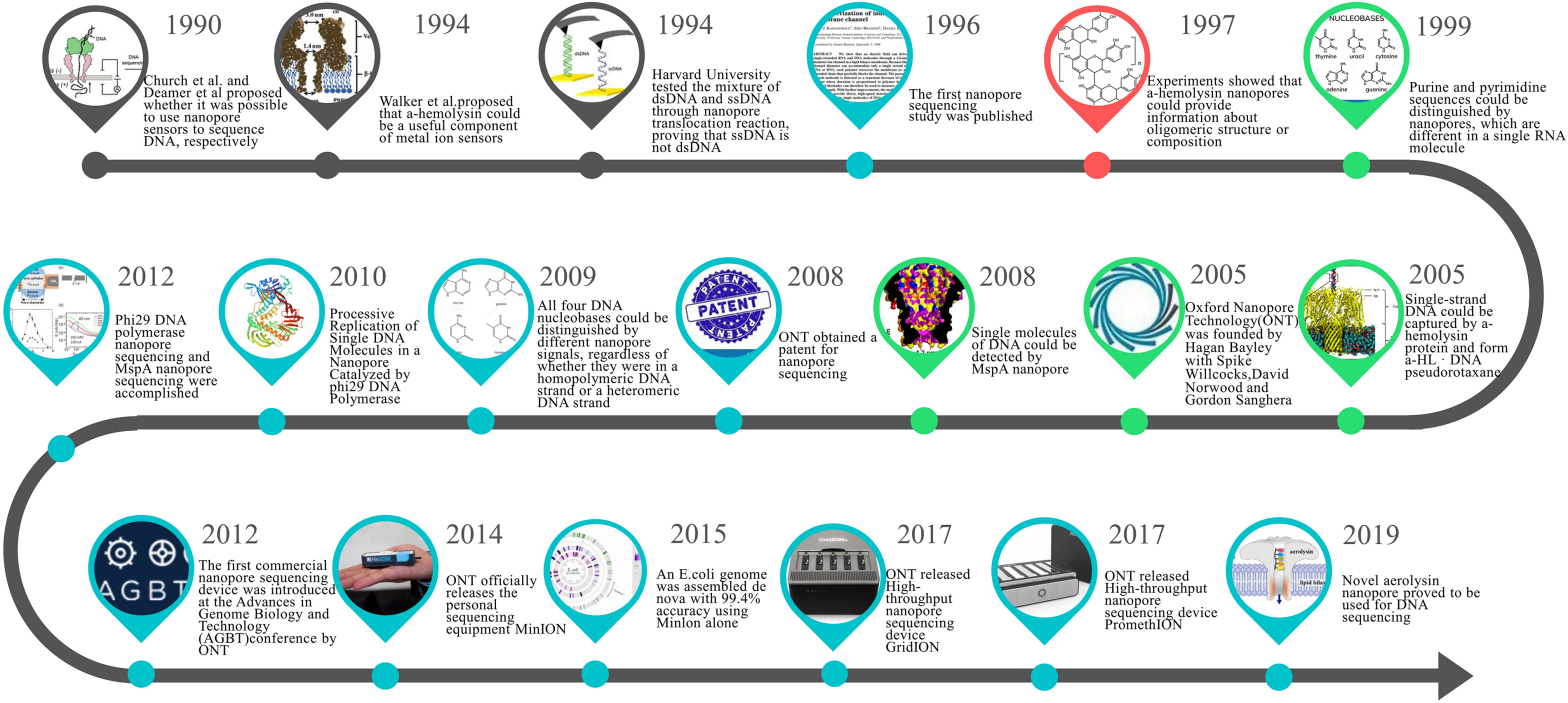
Milestones in the development of nanopore sequencing technology (NST).

Bailey joined his alma mater Oxford University as a professor in Chemical Engineering in 2003, where he founded a company later called Oxford Nanopore Technologies (ONT) in 2005 and organized an experienced development team to commercialize NST. In 2008, ONT obtained a core nanopore sequencing patent and subsequently released the first commercial sequencing device using nanopore technology. Figure 2 summarizes the key time points in developing NST.

## 4. The history of solid-state nanopore sequencing

In 2001, scientists developed a solid-state nanopore manufacturing control method called “ion beam engraving” and used the “ion beam engraving” method to produce nanopores made of Si_3_N_4_, thereby recording a single DNA molecule (Li et al., 2001). This method opened the prelude to DNA sequencing using solid-state nanopore technology. With the advancement of processing technology, solid-state nanopore technology has attracted more and more attention. Solid-state nanopores have some advantages compared with biological ones:

1. Solid-state nanopores have much better chemical and thermal stability, higher durability, and are hard to be denatured; they can keep normal running in extreme environments (Venkatesan et al., 2009);
2. The current pore size of biological nanopores is about 1 ~ 2 nm, while the pore size of solid-state nanopores is generally between 3 ~ 1,000 nm. The relatively large nanopore size enables solid-state nanopores to detect DNA and study proteomics and pathogen screening at the same time (Tsutsui et al., 2019);
3. Solid-state nanopores have advantages in reducing manufacturing difficulty and cost, which can be modified according to users’ needs (Roman et al., 2018). There are studies investing efforts in cost reduction to ensure the performance of solid-state nanopores, and good results have been achieved. Several main materials used to prepare solid-state nanopores are as follows: silicon nitride (Si_3_N_4_), silicon dioxide (SiO_2_), alumina (Al_2_O_3_), boron nitride nanopores (BN), graphene, polymer films, and hybrid materials (Feng et al., 2015). In recent years, solid-state nanopore technology has gradually improved. For example, it has been found that Si_3_N_4_ solid-state nanopores can provide the highest signal-to-noise ratio (SNR). Compared with MspA, solid-state nanopores can increase SNR by >160 fold (Fragasso et al., 2020), which may indicate the future development direction of NST. Intrinsic background fluorescence is generated during the preparation of solid-state nanopores, which can affect signal readout accuracy. Background fluorescence is inevitable, but how to reduce its influence is a research hotspot. Some studies have suggested that the background fluorescence of silicon nitride films can be reduced by using a focused helium ion beam, and the SNR of signal reading can be improved (Sawafta et al., 2014); Research has also been conducted on improving the properties of nanopores in the solution since proteins pass through nanopores too fast to be accurately detected. Some researchers have found that they can slow down protein nanoparticles and increase their residence time within the nanopore by placing hydrogels, thereby improving signal resolution. The dwell time in the medium increases the detection rate (Acharya et al., 2020). At present, on the one hand, most of the studies focus on improving the performance of solid-state nanopores. On the other hand, there are increasing studies aiming to expand the application scope of solid-state NST in areas such as detecting single-molecule DNA (Goto et al., 2020), investigating the mechanical properties of liposomes (Lee et al., 2018), analyzing macromolecular peptide biomarkers (Chau et al., 2020) and protein–protein conformation and interaction (Chae et al., 2018), exploring tetrahedral DNA nanostructures (TDN) (Zhao et al., 2019) and others.

## 5. Types of nanopore sequencing

Nanopore sequencing technologies can be divided into biological nanopores and solid-state nanopores.

### 5.1. Biological nanopores

#### 5.1.1. α-Hemolysin

α-Hemolysin (α-HL) is the first bio-nanopore to be applied in practice, and it is also the absolute pillar of the early development of NST. α-Hemolysin (α-HL), secreted by Staphylococcus aureus as a 33.2 kD water-soluble monomer, can self-assemble to form a 232.4 kD heptamer transmembrane channel, and form a cap with a diameter of 3.6 nm and a 2.6 nm diameter transmembrane b-barrel constitutes the protein nanopore channel (Song et al., 1996). The nanopore size is 10 nm × 10 nm, and the narrowest part is 1.4 nm. The inner diameter of the channel is very close to the size of the ssDNA molecule (1.3 nm in diameter). Therefore, the α-HL nanopore can accommodate the passage of ssDNA and distinguish individual nucleotides by changing the ion current in the nanopore (Cherf et al., 2012). Therefore, α-HL has become a very promising material in nanopore sequencing. Moreover, the α-HL nanoporous structure can be functionally stable at a pH of 2–12 at a temperature close to 100°C (Kang et al., 2005). These factors ultimately determine that α-HL becomes the first material for commercial applications of nanopore sequencing equipment.

#### 5.1.2. MspA

Mycobacterium smegmatis porin A (MspA) is a protein nanopore with excellent properties and broad application prospects (Branton et al., 2008). It is also the main application material of the current Oxford Nanopore Technologies (ONT) sequencing equipment. MspA has a similar mechanism to α-HL and can better bind metal ions to stabilize the structure of the protein. In addition, the ionic current generated by ssDNA passing through the MspA nanopore has a greater difference and a clearer signal. MspA is an octamer with a minimum internal diameter of 1 nm, narrower than α-HL’s channel but more stable, so it can improve the signal resolution of DNA sequencing and has better structural stability under extreme conditions than α-HL. Therefore, it is very suitable for responding to emergencies (Abiola et al., 2003). A study on the structure of MspA has fully demonstrated the potential and possibility of MspA as a nanopore sequencing material (Derrington et al., 2010).

#### 5.1.3. Bacteriophage phi29

The previous NST was named based on the materials used, though this present technology is named after phi29 DNA polymerase (phi29 DNAP). Phi29 DNAP has a good sustainable synthesis ability and a high affinity for DNA substrates (Lieberman et al., 2010). The phage phi29 DNA packaging motor is a dodecamer portal protein complex that uses a hexamer packaging RNA (pRNA) to drive the protein. The pRNA has strong self-assembly properties, making the entire complex structure highly sensitive (Wang et al., 2013). In the middle of the structure composed of 12 protein subunits, a 3.6 nm channel allows dsDNA to pass through the middle (Haque et al., 2018), which contrasts with the disadvantages of small pore size in most biological nanopores studied in the past. The phage phi29 DNA packaging motor is a nanopore with a larger diameter, the narrowest at the N-terminus being 3.6 nm and the widest at C-terminus being 6 nm; the larger diameter endows MspA with the possibility to detect macromolecular biomarkers (Ji et al., 2016). At the same time, the study found that the length of the phage phi29 DNA packaging motor is about 7 nm, and the cross-sectional area of the middle nanopore channel is between 10 and 28 nm^2^ (Xiang et al., 2006). A better understanding of the translocation dynamics of the phi29 system could lead to the development of more advanced DNA translocation systems (Haque et al., 2015), which could allow us to modify and improve protein nanopores from a protein engineering perspective to give them new detection capabilities.

#### 5.1.4. Aerolysin nanopore

Aerolysin is a channel-forming toxin secreted by *Aeromonas* spp. The monomer of aerolysin can undergo a concentration-dependent transition to become a mushroom-shaped heptameric protein that is insertion-competent (Rossjohn et al., 1998). Aerolysin nanopores have a structure very similar to α-HL nanopores, and the channel diameter of aerolysin nanopores is 1.0–1.7 nm, which is smaller than that of α-HL nanopores (Degiacomi et al., 2013). Smaller nanopores produce more stable signals when passing through dsDNA. These conditions make aerolysin a superior choice for nanopore sequencing materials. According to reports in the literature, studies on using aerolysin nanopores for oligonucleotide identification are ongoing (Cao et al., 2016). Furthermore, the aerolysin nanopore also shows its potential in peptides/protein sequencing. Studies have shown that 13 of the 20 natural amino acids can be identified with the help of short polycation carriers that single-molecule traps can restrict in the aerolysin sensing area (Ouldali et al., 2020).

### 5.2. Solid-state nanopore

#### 5.2.1. Atomically thin 2D nanopore

Recently, the advancement of materials science has brought new possibilities to the construction of nanopores. Especially, 2D materials have attracted particular attention for their ultra-thin thickness. Graphene, a single-layer allotrope of carbon, is considered to have the potential to achieve single-base resolution due to its atomic thickness and excellent electromechanical properties (Garaj et al., 2010). However, there are still several defects in using graphene as a nanopore sequencing material. In the experiments, graphene nanopores were blocked due to the specific hydrophobic interaction between DNA and graphene. To solve this problem, a new self-assembled monolayer pyrenediol was invented, which can change the hydrophobicity of graphene to prevent nanopore clogging (Schneider et al., 2013). Another restriction of the graphene nanopore is the signal-to-noise ratio. 1/f noise is higher in monolayer and bilayer graphene nanopores than in silicon nitride nanopores. Therefore, multiplying the graphene layer would decrease the noise level and increase the membrane thickness (Heerema et al., 2015). Molybdenum disulfide (MoS_2_) is another atomically thin two-dimensional material with sub-nanometer thickness, showing a better signal-to-noise ratio in past studies (Liu et al., 2014). At the same time, some studies have found that the MoS_2_ nanoporous membrane is a very good nanoporous material, which has the same excellent performance as graphene nanopores in many aspects, such as spatial resolution and lateral detection performance.

#### 5.2.2. Single-walled carbon nanotube

As a type of multifunctional nanomaterial, SWCNT has been thoroughly developed and widely applied in various fields due to its excellent electromechanical properties, multiple modification sites, and high biocompatibility (Nag et al., 2021). These advantages also make SWCNT a possible material for making nanopores. According to literature reports, the first SWCNT nanopores were fabricated by inserting ultra-short SWCNTs into lipid bilayers. And when DNA translocation behavior in SWCNT nanopores varies, changes in ionic current can be detected (Liu et al., 2015). In addition, studies have shown that the single ion transport of four different amino acid cations has been examined through the SWCNT with a diameter of 2.25 nm, and it has been found that the conductivity change and mobility measurement using SWCNT has the potential to identify specific amino acids (Ellison et al., 2017).

## 6. Applications of nanopore sequencing

### 6.1. Application of nanopore sequencing in POCT

Point-of-care testing (POCT) is normally performed away from the laboratory with portable equipment; it is also known as rapid testing or near-patient testing (Ferreira et al., 2018). Performing the test directly next to a patient will help provide quick feedback on the results. The POCT does not require transporting test samples, and the analysis process is simplified; it does not necessarily require laboratory staff to perform the test; patients, family members, nurses, etc., can do it. Rapid test results can be used for screening, monitoring or diagnosis. POCT is expected to play more important in places such as hospitals, emergency rooms, specialist clinics, or ambulances and can also be used for patients to complete a self-test at home. The new generation of NST requires fewer samples, small size, convenient carrying, simple operation, and can complete sequencing in various complex environments, meeting the needs of POCT.

Remote areas far from laboratories often have more epidemic prevention and control difficulties. Ebola virus (EBOV), the ongoing outbreak in West Africa that began in 2014, is a typical example; during the Ebola outbreak in West Africa, researchers at the Field Diagnostics Laboratory in Liberia successfully used a new pocket-sized nanopore sequencer (Hoenen et al., 2016). This technology has the advantages of convenience and speed. It only takes 3 h from the sample to identify the Venezuelan equine encephalitis virus and Ebola virus, which proves that POCT can be powerful in detecting RNA virus on-site (Kilianski et al., 2016). Cancer can occur anywhere in the human body, and the pre-cancer is often very insidious, so the early detection of cancer is very important, but the current detection of cancer recurrence and monitoring after cancer treatment lack rapidity and timeliness. The research reports that detecting structural variation based on nanopore sequencing can achieve individualized disease monitoring based on circulating tumor DNA in cancer patients. This method can increase the sensitivity of disease monitoring to a level where smarter treatment methods can be envisaged (Valle-Inclan et al., 2021). In addition, the researchers found that portable NST has the potential for rapid molecular diagnosis in cancer and proposed using nanopore sequencing to accelerate comprehensive diagnosis and improve patient care (Euskirchen et al., 2017). Nanopore sequencing may become an excellent tool for low-level detection of molecular recurrence, early detection, or cancer-related structural variations required for treatment monitoring (Norris et al., 2016).

Genome analysis is a typical clinical trial. NST has proven to be an effective tool for TP53 mutation monitoring (Minervini et al., 2016). One study shows the MinION device can identify pathogens from samples and obtain resistance genes from urine in about as long as PCR sequencing (Schmidt et al., 2017). The combined use of nanopore sequencing and isothermal amplification offers the possibility of developing timely assays for other viruses (McNaughton et al., 2019). The study also showed that even under limited conditions, the nanopore sequencing device MinION, combined with isothermal amplification, performed well in identifying human malaria parasites (Imai et al., 2017). Experiments show that real-time genome sequencing of clinical isolates takes less time than phenotypic susceptibility testing (Lemon et al., 2017). Nanopore sequencing results also show that detecting Mycobacterium tuberculosis from the clinic takes less than 24 h (Lee and Pai, 2017).

More recently, the combined use of portable nanopore sequencing equipment with simplified sample preparation protocols has opened up the possibility of DNA sequencing in microgravity environments (McIntyre et al., 2016). The MinION nanopore sequencing device has demonstrated its versatility in a variety of harsh environments, and the subsequent miniaturization of the sequencing platform (SmidgION) and the advent of associated library preparation tools (Zumbador, VolTRAX) have further refined nanopore sequencing for DNA sequencing applications and RNA-sequencing applications (Jain et al., 2016).

### 6.2. Application of nanopore sequencing in plant genome

Since the first plant genome of Arabidopsis was deciphered in 2000 (The Arabidopsis Genome Initiative, 2000), DNA sequencing has always been a decisive step in discovering new insights in biology. Plant genomes often have a lot of repetitive sequences, and the genome size is relatively large. The long-read characteristics of nanopore sequencing applied to plant genome sequencing make it easier to assemble high-quality genome sequences (Murigneux et al., 2020). As an emerging technology, nanopore sequencing has found many meaningful applications in plant genome sequencing. There are research reports that use the combination of Illumina and Nanopore sequencing platforms to provide the first chromosome-level genome data of Taxus Chinensis and construct the whole genome phylogeny. The tree lays the foundation for improving the excellent genetic traits of Taxus Chinensis in the future (Ning et al., 2020). Structural variation is the basis for improved crop traits. Scientists have applied nanopore sequencing to detect crop structural variation and successfully captured 238,490 structural variations in more than 100 different tomato strains. And through the combination of quantitative genetics and genome editing, researchers have shown how to change the structural variation of genes and expression levels to affect the yield, size and other traits of tomatoes, which has guiding significance for the improvement of excellent traits of tomatoes (Alonge et al., 2020). In another study, scientists used nanopore sequencing equipment to sequence the whole genomes of two basmati rice varieties, providing material for subsequent genomic and functional analyses (Choi et al., 2020). mRNA methylation (m6A) mutation is an important research content of epigenetics. It refers to the modification and change of 6-methyladenosine (m6A) that occurs on mRNA molecules, regulating gene expression and splicing. RNA stability and other aspects play an important role. Studies have reported using nanopores to perform direct RNA sequencing of wild-type and mRNA methylation mutants from the model plant Arabidopsis thaliana, which shed light on the complexity of mRNA processing and modification in long single-molecule reads that could contribute to the refinement of Arabidopsis genome annotation (Parker et al., 2020). The short-read sequencing technology represented by Illumina often results in incomplete and fragmented results due to plant genomes’ highly reproducible and polyploid nature. Therefore, the researchers developed a protocol based on long-read sequencing devices (MinION or PromethION sequencers) and optical mapping (Saphyr systems); and they used this protocol to generate high-quality genomes for two new dicot morphological types Brassica Z1. The sequence proved its effectiveness (Belser et al., 2018).

### 6.3. Application of nanopore sequencing in the bacterial genome

The difference between bacterial phenotypes is tiny, and the size of the bacteria is often very small, which is difficult to monitor. Therefore, it is vital to type bacteria through whole-genome sequencing technology. Scientists from NASA’s Environmental Health System (EHS) use NST to sequence microbes collected and cultured on the International Space Station(ISS)and transmit the results to the Earth. Then, the same samples on the Earth are sequenced in a standard laboratory to confirm the identity of microbes samples. This marks the first time microbes have been sequenced completely outside the Earth (Burton et al., 2020).

MinION™ DNA sequencer is valuable for both high taxonomic resolution and microbial diversity analysis. This platform has been used for many studies in this field. For instance, amplifiers of bacterial 16S rRNA gene have been sequenced, and the data obtained are enough to reconstruct more than 90% of 16S rRNA gene sequences for 20 species in the simulated reference community (Benítez-Páez et al., 2016). An approach has been developed to using the MinION ™ DNA sequencer’s long reading ability to quickly sequence the “rrn” ribosomal operators (metagenomics) of complex natural communities. Due to the small and convenient platform, the possibility of characterizing the microbiota on-site or on the robot platform has become a reality (Kerkhof et al., 2017). MinION ™ DNA sequencer may also provide more accurate distribution. The platform breaks through the limitations of short reading to determine the composition of the macro genome during the shotgun-based whole DNA sequencing (metagenomics), and obtains the sequencing result of root sign (Brown et al., 2017). At the same time, researchers use a MinION ™ DNA sequencer to perform long amplicon sequencing. The obtained data are sufficient to study two mock microbial communities in a multiplex manner and to almost wholly reconstruct the microbial diversity of these two mock communities (Benítez-Páez and Sanz, 2017).

The Illumina sequencing platform has been widely used, but the short read length limits its ability to be applied to the genome, while nanopore sequencing has the advantage of a long read length that makes up for this deficiency. A study showed that 12 strains of Klebsiella pneumoniae were sequenced using the MinION sequencing equipment, and combined with the traditional Illumina sequencing results, good sequencing results were obtained (Wick et al., 2017). Other researchers also conducted similar experiments to sequence the genome of Rickettsia typhi isolates, using v9.5 chemical preparation and sequencing libraries on MinION in Vientiane, Laos, and on the Illumina sequencing device of a British laboratory. The same isolates as above were sequenced. Sequence reads from the nanopore sequencing device MinION versus reads from the Illumina device show that the frequency of false-positive errors in MinION results is too high (Elliott et al., 2020). Mantas Sereika sequenced activated sludge from an anaerobic digester using single runs of Illumina MiSeq 2 × 300 bp, PacBio HiFi, and Oxford Nanopore R9.4.1 and R10.4 and showed that Oxford Nanopore R10.4 could be used to generate near-finished microbial genomes from isolates or metagenomes without short-read or reference polishing.

Traditional microbial freshwater testing focuses on detecting specific bacterial indicator species, and sequencing all microbial DNA through metagenomics is an effective method. Studies have reported using the deterministic composition and spatiotemporal microbiota of the surface water from the Cambridge river to provide optimized experimental and bioinformatics guidelines. It is found that the application of NST in genomics can describe the hydrological core microbiome and fine time gradient, which is in line with complementary physicochemical measurements (Urban et al., 2021). There are also studies reporting that the whole genome sequencing of the Bacillus anthracis nanopore from human anthrax isolates was completed several hours later in a highly enclosed laboratory (McLaughlin et al., 2020). Another similar study used sequencing equipment from Illumina and from Oxford Nanopore Technologies (ONT) or SMRT Pacific Biosciences (PacBio) to compare mixed assemblies of 20 bacterial isolates, including two reference strains. Combining ONT and Illumina reads in the genome assembly process promotes high-quality genome reconstruction (De Maio et al., 2019). Metagenomic sequencing can more quickly identify bacterial lower respiratory infections (LRI) pathogens in the clinic and provide an important reference for clinical diagnosis, but this method needs to remove a large amount of human DNA in these samples. To this end, the researchers developed a method for identifying bacterial LRIs based on NST, which was experimentally validated. The method was first tested on 40 samples and then optimized on another 41 samples, and the optimized method can accurately detect antibiotic resistance genes (Charalampous et al., 2019).

### 6.4. Application of nanopore sequencing in the viral genome

NST has a unique advantage in real-time virus monitoring, virus evolution, and genetic mutation research due to its ultra-long read length that can cover most of the viral genome and convenient and fast sequencing process, and it has since been widely used. Compared with the next-generation sequencing (NGS) platform, nanopore sequencing can obtain the whole genome for many viruses without using any assembly algorithms, which can avoid artifacts and errors. Additionally, the long reads from nanopore sequencing can reveal the insertion of the virus’s genome segments into the host genome, such as the integration events of HBV and HPV in the human genome, which bring new insights into the mechanism of tumorigenesis related to viruses. The genome sequencing process for the dengue virus takes a long time, is costly, and relies on PCR technology. To this end, the researchers developed a comprehensive molecular sequencing method for the complete dengue virus genome, using nanopore technology for sequencing while using bioinformatic tools (Nano-Q) to distinguish virus variants in the host. The results showed that under the premise of coverage >100, the dengue virus genome sequence obtained by this method and the counterpart generated by Illumina have a pairwise sequence similarity of more than 99.5%. And the maximum likelihood phylogenetic tree generated from the consensus sequence of the nanopore can reproduce the accurate tree generated by Illumina sequencing with a conservative 99% guide threshold (after 1,000 repetitions and 10% aging) (Adikari et al., 2020). Since the pathology study of hepatitis B virus (HBV) is based on full-length hepatitis B virus genome structure data, researchers have developed a protocol that uses an isothermal rolling circle to amplify HBV DNA while using nanopore technology for sequencing. This protocol also provides potential options for the early detection of HBV and other viruses (McNaughton et al., 2019). Seneca virus A (SVA) has been identified as the cause of vesicular disease in different countries, but we lack the knowledge of prevention and diagnosis based on the sequence structure of the virus. Therefore, scientists performed direct RNA sequencing and PCR-cDNA sequencing on the SVA model using the MinION nanopore sequencing equipment and then assembled the whole genome sequence. Consistent accuracies after analytical optimization were 94 and 99%, respectively. It provides a reference for improving the understanding and discovery of SVA and the prevention of other infectious viruses (Tan et al., 2020). Studies have reported that direct cDNA sequencing of the hepatitis A virus (HAV) using ONT sequencing equipment can obtain the entire HAV genome (Batista et al., 2020). Peste des petits ruminants virus (PPRV) is a virus that Sinopharm focuses on and controls. At the same time, PPRV exists in many remote and backward areas. These laboratories lack the conditions for high-throughput sequencing. To this end, some researchers have developed a new whole-genome sequencing PPRV protocol using portable miniPCR and MinION. This protocol has successfully extracted the complete genome from cell cultures and naturally infected goat samples (Torsson et al., 2020). The traditional short-read (Illumina) sequencing method is difficult to achieve good sequencing results due to amplification and recoding in virus direct RNA sequencing, while the long-read NST makes up for these shortcomings. Some researchers used herpes simplex virus (HSV)-infected primary fibroblasts as a template to provide guidelines for using Oxford Nanopore Technology to construct a direct RNA sequencing library, which provides an important reference for other researchers to conduct experiments (Depledge and Wilson, 2020). The Ebola virus spreads on a large scale, has a very high lethal rate, and mutates rapidly. Data show that the Ebola virus (EBOV) genome replacement rate in the Makona strain is estimated to be 0.87 × 10 (−3) per site per year. and 1.42 × 10 (−3) mutations. Therefore, long-term monitoring of the Ebola virus by genome sequencing has become the top priority in the prevention and treatment of the Ebola virus. Some researchers developed a genome monitoring system using NST and conducted experiments in Guinea in 2015, producing results within less than 24 h after receiving a positive Ebola sample. This shows that it is possible to establish real-time genome monitoring in remote and backward areas, providing an important reference for monitoring the Ebola virus and other infectious viruses (Quick et al., 2016).

### 6.5. Application of nanopore sequencing in the detection of SARS-CoV-2

Severe acute respiratory syndrome coronavirus 2 (SARS-CoV-2) is the culprit behind the coronavirus disease (COVID-19) pandemic that started in 2019 and continues today. Accurate detection of the SARS-CoV-2 virus using genetic sequencing equipment is critical for epidemic prevention and control and for tracking the evolution of the SARS-CoV-2 virus to produce vaccines (Chan et al., 2020). Nanopore sequencing can be used in various environments, and its advantages of convenience and speed make nanopore sequencing widely used to detect COVID-19 and other respiratory viruses. During the COVID-19 pandemic in India in 2020, the National Center for Disease Control (NCDC) of India performed whole genome sequencing of 104 COVID-19 patients using the MinION nanopore sequencing device, which played an important auxiliary role in epidemic prevention and disease control (Kumar et al., 2020). During the COVID-19 pandemic in the UK in March 2020, researchers used the MinION nanopore sequencing device to sequence 1,000 COVID-19 samples from Cambridge hospitals in the UK, yielding 747 high-quality genomes. This approach allows us to effectively monitor the invisible SARS-CoV-2 virus. It has also been demonstrated that combining nanopore sequencing and PCR sequencing technology can effectively evaluate the binding capacity in clinical tests (Meredith et al., 2020). Moore et al. also conducted a similar study in the UK and achieved good results (Moore et al., 2020). During the COVID-19 pandemic in September 2020, the MinION nanopore sequencing device was also used to prevent and control this outbreak. A retest of 619 discharged COVID-19 cases found that 87 were retested positive for SARS-CoV-2 (Lu et al., 2020). During the year 2020 COVID-19 outbreak in the United States, researchers used ONT nanopore sequencing equipment to perform metagenomic sequencing of NP swab specimens from 50 COVID-19 patients and detected a decrease in the diversity of microbial communities in these patients, suggesting the usefulness of NST for metagenomic sequencing of SARS CoV-2 NP swabs (Mostafa et al., 2020). The read length of nanopore sequencing is longer than that of previous generations of sequencing technologies, and it has advantages in turnaround time, portability, and cost, making nanopore sequencing widely used in the frontline of the fight against COVID-19 outbreaks in various countries. However, the limited sequencing accuracy of nanopore sequencing also causes concern about the application of nanopore sequencing to monitor SARS-CoV-2. Therefore, the researchers performed viral whole-genome sequencing on 157 SARS-CoV-2 patient samples and synthetic RNA controls using ONT nanopore sequencing equipment and Illumina conventional sequencing equipment, respectively. The results showed that the ONT sequencing results had a higher error rate than the Illumina device but still achieved highly accurate consensus-level sequencing. At the same time, the ONT sequencing facility has also uncovered new information on structural diversity (Bull et al., 2020). A similar study was conducted by researchers using traditional Illumina sequencing equipment and nanopore sequencing equipment to perform SARS-CoV-2 whole-genome sequencing (WGS) to evaluate performance (Charre et al., 2020).

### 6.6. Application of nanopore sequencing in direct RNA sequencing

Direct RNA sequencing does not require reverse transcription and amplification, reducing the possibility of errors due to reprogramming and mismatches. Direct RNA sequencing can characterize cell morphology, reveal viral genome information, determine cell gene expression status, and detect post-transcriptional alternative splicing differences and RNA modifications. And the long-read, convenient, and fast NST is very suitable for direct RNA sequencing. Researchers have developed a direct RNA-seq sequencing method based on NST, which is a highly parallel, real-time, single-molecule method that does not require reverse transcription and amplification, has long read lengths, and can generate full-length, strand-specific RNA sequences (Garalde et al., 2018). Single-molecule N6-methyladenosine (m6A) detection is an important part of RNA sequencing, but there is currently no comprehensive method for direct detection. To this end, the researchers developed a new method, “Nanom6A,” which uses nanopore technology direct RNA sequencing of samples based on the XGBoost model, that can achieve single-base resolution and quantitative analysis of m6A modifications (Gao et al., 2021a). Understanding genome organization and gene regulation require an in-depth understanding of RNA transcription, processing, and modification. Some researchers have used nanopores to directly sequence Arabidopsis wild-type and mRNA methylation-deficient (m6A) mutant RNAs. The results show that direct RNA sequencing using NST can reveal the complexity of mRNA processing and modification in target genes, and these findings help further refine the Arabidopsis genome annotation (Parker et al., 2020). Some researchers used nanopore direct RNA sequencing technology to detect N6-methyladenosine (m6A) RNA modification with an accuracy rate of 90%. These results initially revealed the application prospects of NST in the field of direct RNA sequencing (Liu H. et al., 2019).

### 6.7. Progress of nanopore technology in the field of protein sequencing

Nanopore technology shows great versatility in biological analysis. It can analyze samples quickly and conveniently and, in addition, has the possibility of modifying and improving the nanopore structure to meet more needs. In recent years, with the development of nanopore sequencing equipment and the improvement of supporting tools, nanopore sequencing equipment has increasingly demonstrated its versatility in detecting various biomolecules. In DNA sequencing especially, the ONT has subsequently launched a variety of commercial nanopore sequencing equipment. The future development direction of nanopore sequencing is to apply NST to detect proteins and peptides that play a direct role in life activities.

According to the current research status, sequencing proteins and peptides is very challenging work. At present, we mainly face three difficulties: (1) How to unfold the protein structure safely and effectively. Protein has a larger molecular weight than DNA and RNA, the current nanopores are too small, the protein structure is complex, and the charge density distribution is uneven. Therefore, if we want to detect and analyze a protein, we need first to untie the high-level structure of the protein and make it a chain of amino acids that can pass through nanopores; (2) Peptides are recognized by nanopore signals. The aim is to ensure that the amino acid chain passes through the nanopore at a stable and appropriate speed, which produces a sufficiently recognizable difference in ion current changes. Unfortunately, the current research cannot control the speed of the amino acid chain through the nanopore to reach the commercial level; (3) Amino acid identification. This last step is to distinguish amino acids based on the difference in the ionic current generated by the amino acid chain through the nanopore. At the same time, various modifications may be distributed on the polypeptide chain, such as methylation, acetylation, phosphorylation, and more. These structures affect the resolution of protein sequencing.

In recent years, researchers have conducted many studies applying NST to protein sequencing, and a series of advances have been made. Studies have shown that protein sequencing using aerolysin nanopores has found that the difference in ionic current changes induced by the passage of 20 natural amino acids through nanopores is far from the level that can be detected by current equipment. To this end, the researchers proposed to use the σ′ value to increase the conductivity of the solution. The σ′ value describes the comprehensive result of ion mobility in the nanopore, which helps to provide further insights for nanopore protein sequencing (Huo et al., 2021). Another study reported that the E. coli protein OmpG has a single polypeptide chain, which is also an ideal substitute for nanopore protein sequencing, which enriches the choice of nanopore protein sequencing materials (Sanganna Gari et al., 2019).

Traditional nanopore sequencing, such as DNA sequencing, relies on the uniform linear charge density of DNA. However, proteins and peptides with complex structures often have heterogeneous charge densities, making it uncertain for them to perform nanopore translocation. To this end, the researchers developed a direct, model-free, real-time single-molecule method for monitoring the translocation of disordered heterogeneous charged peptides through nanopores. The two “selective tags” at the end of abortion were mainly used to prove the translocation of peptides’ high sensitivity to applied transmembrane potential (Hoogerheide et al., 2018). The selection of nanopore sequencing materials is the key. The feasibility of protein sequencing was tested by molecular dynamics simulation of graphene nanopores, and the single-chain phenylalanine glycine repeat peptide was taken as the sample. It is found that peptides will adhere to graphene and show when subjected to transmembrane deviation or hydrostatic pressure gradient. As translocation occurs, the difference in ionic current changes generated during nanopore transport is altered by the type of amino acid passing through the nanopore. The authors initially verified the feasibility of using graphene materials for protein nanopore sequencing (Wilson et al., 2016). The study also reported the method of protein sequencing using trichromatic fluorescence and plasma nanopore equipment. Computer simulation results show that this method can correctly identify most proteins in the human proteome. Moreover, even considering the actual experimental conditions, the deep learning protein classifier can reach 97% of the whole proteome accuracy. Applying this method to clinically relevant protein data sets, a correct protein recognition rate of about 98% was obtained (Ohayon et al., 2019). In addition to developing new nanopore protein sequencing materials, the use of existing materials for improvement is also an important direction for developing protein sequencing. Studies have reported using balanced all-atom molecular dynamics simulations to estimate the changes in the α-hemolysin nanopore signal associated with 20 standard amino acids. The results showed that the current α-hemolysin was affected by the amino acid’s volume, hydrophobicity, and net charge and could not generate a signal with sufficient resolution. Based on this result, an improved method for modifying α-hemolysin nanopores was proposed (Di Muccio et al., 2019).

## 7. Bioinformatics of nanopore sequencing

At the initial launch of ONT’s commercial nanopore sequencing equipment MinION, the company did not provide supporting sequence analysis tools, while all the other tools available then could not process the equipment’s special output data format FAST5. In view of this, ONT developed a Poretools Toolkit, which can directly manipulate the native FAST5 file format, as well as provide a range of format conversion, data exploration, and visualization tools (Kono and Arakawa, 2019).

At the same time, another new problem appears. The analysis tools developed based on traditional sequencing methods can no longer meet the needs of analyzing the results produced by nanopore sequencing equipment. Therefore, researchers have worked hard to develop new tools for various purposes. The tables given in the following discussions are incomplete, but we try to introduce as many representative tools as possible that can be applied to the analysis of nanopore sequencing results.

### 7.1. Base calling

Base recognition is the initial step in any sequencing method. In nanopore sequencing, when a D strand passes through the nanopore, the current of the nanopore changes, and the current change caused by different bases is also different. Base recognition converts the nucleotide sequence (A, C, G, T) readings from the electrical signal changes collected from the nanopore sequencing instrument (MinION, GridION, or PromethION). A series of advances in base calling analysis based on nanopore sequencing have been made in recent years. Albacore, Guppy, and Chiron are the classic basecaller tools. Regarding accuracy metrics, ONT’s Albacore and Guppy are superior, while the third-party tool Chiron does not perform well. As far as speed is concerned, Guppy is the fastest due to its GPU acceleration, while Chiron is the slowest, despite using GPU acceleration as well (Wick et al., 2019). In addition, Bonito, an open-source research project offered by ONT, completes the training and basecalling process. Bonito is primarily a GPU-focused project that offers significant improvements in raw accuracy but at the expense of a slower base finding algorithm. You can find the open-source code for this method in the table at the end of the section if you are interested in its development.

Recently, it has been a promising direction to use deep learning and neural network technology to analyze nanopore sequencing data. Some researchers have proposed an improved U-net model to transform the previous typical sequence tag-based calling task into a multi-tag segmentation base calling task. This improvement is based on the union of base calling and segmentation and has achieved competitive results (Zhang et al., 2020). Other researchers have proposed a new deep learning model using a temporary synchronous network (TCN). Compared with the traditional deep learning model of using recurrent neural networks (RNN), TCN has advantages in terms of base calling accuracy and speed (Zeng et al., 2020). According to the current deficiencies and actual needs, researchers have developed various new algorithms and tools with various functions and advantages. EpiNano, an algorithm for predicting m6A RNA modification from dRNA sequence data sets, can now train models with features extracted from both basecalled dRNA seq FASTQ data and raw FAST5 nanopore outputs (Liu H. et al., 2021). Ravvent is a new basecaller that uses joint processing of raw and event data and is based on an encoder-decoder architecture of recurrent neural networks (Napieralski and Nowak, 2022). NanoReviser, an open-source DNA basecalling reviser, is based on a deep learning algorithm to correct the basecalling errors introduced by current basecallers provided by default (Wang et al., 2020). DeepNano coral achieves real-time base calling during sequencing with an accuracy slightly better than the fast mode of the Guppy base caller and is extremely energy efficient, using only 10 W of power (Perešíni et al., 2021). Sigmap, a nanopore raw signal mapper tool, can map raw nanopore signals for real-time selective sequencing and has considerable performance in drawing yeast-simulated original signals (Zhang et al., 2021). UNCALLED is an open-source mapper that rapidly matches the streaming of nanopore current signals to a reference sequence (Kovaka et al., 2021). There are many new studies on analysis tools, and some have been proposed to meet the increasing demand for base calling computing. A custom FPGA that operates in tandem with a CPU across a high-speed serial link and a simple API has achieved a measured speed-up over CPU-only basecalling over 100× with an energy efficiency improvement of three orders of magnitude (Wu et al., 2020). Readfish, a toolkit, can use GPU to selectively sequence certain DNA molecules in a pool, enabling enumeration and deletion to address biological questions (Payne et al., 2021). We have also found the mainstream base callers for nanopore sequencing (Edwards et al., 2019; Mitsuhashi et al., 2019; Shabardina et al., 2019). The characteristics of each of these base callers differ, and each is suitable for use in a specific situation. Moreover, some of them are out of date due to technological advancements, but they can still witness the development of NST. Below is a description of the software used for base calling:

**Table 1:** 

Tool	Availability
Albacore	https://github.com/nanoporetech/albacore
BasecRAWller	Not Available
Bonito	https://github.com/nanoporetech/bonito
Buttery-Eel	https://github.com/Psy-Fer/buttery-eel
C3Poa	https://github.com/rvolden/C3POa
CATCaller	https://github.com/biomed-AI/CATCaller
Causalcall	https://github.com/scutbioinformatic/causalcall
Chiron	https://github.com/haotianteng/Chiron
DeepNano	https://bitbucket.org/vboza/deepnano/src/master/
DeepNano-Blitz	https://github.com/fmfi-compbio/deepnano-blitz
Deepore	https://github.com/etheleon/deepore
Dorado	https://github.com/nanoporetech/dorado
Flappie	https://github.com/nanoporetech/flappie
Guppy	Only to ONT customers
Halcyon	https://github.com/relastle/halcyon
Katuali	https://github.com/nanoporetech/katuali
Megalodon	https://github.com/nanoporetech/megalodon
MicroPIPE	https://github.com/BeatsonLab-MicrobialGenomics/micropipe
MinION Plasmid Sequence Verification Pipeline	https://github.com/scottdbrown/minion-plasmid-consensus
Metrichor	Only to ONT customers
Nanocall	https://github.com/mateidavid/nanocall
NanoDJ	https://github.com/genomicsITER/NanoDJ
Nanonet	https://github.com/ProgramFiles/nanonet
Nanopype	https://github.com/giesselmann/nanopype
Nanoseq	https://github.com/nf-core/nanoseq/
Poreduck	https://github.com/alexiswl/poreduck
PoreOver	https://github.com/jordisr/poreover
Poreplex	https://github.com/hyeshik/poreplex
R2C2	https://github.com/rvolden/C3POa
ReadBouncer	https://github.com/JensUweUlrich/ReadBouncer
Runnie	https://github.com/nanoporetech/flappie/blob/master/RUNNIE.md
SACall	https://github.com/huangnengCSU/SACall-basecaller
Squiggler	https://github.com/JohnUrban/squiggler
Taiyaki	https://github.com/nanoporetech/taiyaki
WaveNano	Not Available

### 7.2. Alignment

Although the overall accuracy of ONT sequencing is gradually improving, the accuracy of some reads is relatively low, and the error rates of 1D reads and 2D / 1D2 reads are high. Therefore, before downstream analysis, self error-correction and hybrid error-correction algorithms are usually used to obtain higher sensitivity and improve the quality of sequencing data.

The algorithm to deal with this problem has been studied for a long time, and the most famous and effective one is the Blast algorithm (Smith and Waterman, 1981). Researchers have developed some sequence alignment tools to solve the specific characteristics of error-prone long reads. Graphmap, the first calibrator designed exclusively for ONT sequencing, was released in 2016. Graphmap can gradually improve candidate comparison to reduce the error rate. While for ~10 kb noisy reads sequences, minimap2 is faster. Minimap2 (Li, 2018), developed by Heng Li of Harvard University, is the most excellent sequence alignment tool, which can perform splicing perception comparison on ONT cDNA or direct RNA sequencing reads. According to the researchers in Liu B. et al. (2019), using graph-based alignment skeletons, they proposed deSALT, which splices reference sequences to provide refined alignments. The DeSALT tool can solve several challenges, such as small exons, serious sequencing errors, and alignments with consensus splices. The method provides a method for producing full-length alignments with a higher level of quality, which has a great deal of potential for transcriptomic research. To accelerate the alignment of sequences with genome graphs, GraphAligner (Rautiainen and Marschall, 2020) was developed, a tool that maps long reads to genome graphs.

In comparison with the previous tools, GraphAligner is 12 times faster. Moreover, its memory usage is five times lower, making it comparable to aligning reads to linear reference genomes. GraphAligner has been found to be almost three times more accurate and 15 times faster than other error correction tools when used to correct errors. As explained in Joshi et al. (2021), QAlign is a preprocessor designed for use with long-read alignment algorithms for nanopore sequencers. Furthermore, it can also be used to overlap long-reads or align RNA-seq reads to transcriptomes in addition to aligning reads to the genome. Despite a similar computational time, QAlign provides more accurate alignments than other alignment programs based on nucleotide sequences. Aligning a sequence to a directed acyclic graph is common in long-read error correction. In light of this, the authors of Gao et al. (2021b) present abPOA (adaptive banded partial order alignment), a SIMD-based C library that uses adaptive banded dynamic programming to achieve fast partial order alignment. Besides its ability to work independently as an alignment tool with consensus calling, it can also be incorporated into any workflow for long-read error correction and assembly. Despite having a similar alignment accuracy to previous tools, abPOA is 15 times faster than previous tools. Following (Fu et al., 2021), Vulcan uses dual-mode long-read alignment to improve the identification of SVs. As a result of variations in mutation rates, Vulcan uses different alignment techniques for different regions of the genome, improving SV detection. SV detection can also be improved by mapping long reads at smaller edit distances with Vulcan. It is also pertinent to note that a wide range of related tools are available, and the following are the most popular software tools that are suitable for aligning long reads of nanopore sequencing:

**Table 2:** 

Tool	Algorithm	Availability
AbPOA	Heaviest bundling algorithm	https://github.com/yangao07/abPOA
Automation	Alignment of nanopore data using HMM	https://github.com/UCSCNanopore/Data
BELLA	Berkeley Efficient Long-Read to Long-Read Aligner and Overlapper	https://github.com/giuliaguidi/bella
Bwa-Sw	Burrows-Wheeler Aligner’s Smith-Waterman Alignment	http://bio-bwa.sourceforge.net/
DeSALT	Long transcriptomic read alignment with de Bruijn graph-based index	https://github.com/hitbc/deSALT
Edyeet	A base-accurate DNA sequence alignments using edlib and mashmap2	https://github.com/ekg/edyeet
FastRemap	A Tool for Quickly Remapping Reads between Genome Assemblies	https://github.com/CMU-SAFARI/FastRemap
GraphAligner	Seed-and-extend program for aligning long error-prone reads to genome graphs	https://github.com/maickrau/GraphAligner
GraphMap	Long read alignment	https://github.com/isovic/graphmap
GraphMap2	splice-aware aligner based on GraphMap	https://github.com/lbcb-sci/graphmap2
Kart	A divide-and-conquer algorithm for NGS read mapping with high error tolerance	https://github.com/hsinnan75/Kart/
LAMSA	Fast split read alignment with long approximate matches	https://github.com/hitbc/LAMSA
LAST	Aligner	http://last.cbrc.jp/
LordFAST	Aligner for long reads	https://github.com/vpc-ccg/lordfast
LRA	Long read aligner	https://github.com/ChaissonLab/LRA
Magic-BLAST	An RNA-seq aligner for long and short reads	https://github.com/ncbi/magicblast
MashMap	An approximate algorithm for mapping long reads to large reference databases	https://github.com/marbl/MashMap
Meta-Aligner	Aligner for long reads	http://brl.ce.sharif.edu/software/meta-aligner
Minialign	Aligner for PacBio and ONT long reads	https://github.com/ocxtal/minialign
Minimap2	Maps reads to a human genome	https://github.com/lh3/minimap2
MinimapR	A parallel alignment tool for the analysis of large-scale third-generation sequencing data	https://github.com/Geehome/minimapR
Mm2-Fast	mm2-fast is an accelerated implementation of minimap2 on modern CPUs	https://github.com/lh3/minimap2/tree/fast-contrib
NanoBLASTer	Aligner for long reads	https://github.com/ruhulsbu/NanoBLASTer
NanoPipe	Provides alignments to any target of interest, alignment statistics and information about polymorphisms	https://github.com/IOB-Muenster/nanopipe2
NGMLR	Long-read mapper designed to align PacBio or Oxford Nanopore (standard and ultra-long) to a reference genome with a focus on reads that span structural variations	https://github.com/philres/ngmlr
NPoRe	An n-Polymer Realigner for improved pileup variant calling	https://github.com/TimD1/nPoRe
Poremap	Pipeline for nanopore read alignment and statistics	https://github.com/camillaip/poremap
QAlign	aligning nanopore reads accurately using current-level modeling	https://github.com/joshidhaivat/QAlign
RawMap	Complements Minimap2 for a fast and efficient Read-Until pipeline	https://github.com/harisankarsadasivan/RawMap
S-ConLSH	alignment-free gapped mapping of noisy long reads	https://github.com/anganachakraborty/S-conLSH-2.0
SelectION	Rapid linking of long reads to a reference genome	https://github.com/giesselmann/selectION
Sigmap	streaming method for mapping raw nanopore signal to reference genomes.	https://github.com/haowenz/sigmap
SmsMap	long-read alignment	https://github.com/NWPU-903PR/smsMap
Starlong	Spliced Transcripts Alignment to a Reference	https://github.com/alexdobin/STAR
SuffixAligner	Long-read aligner	https://github.com/ZeinabRabea/SuffixAligner
ULTRA	A tool for spliced alignment of long transcriptomic reads to a genome, guided by a database of exon annotations	https://github.com/ksahlin/ultra
UNCALLED	Maps raw nanopore signals from fast5 files to large DNA references	https://github.com/skovaka/UNCALLED
Vulcan	A long-read mapping and structural variant calling *via* dual-mode alignment	https://gitlab.com/treangenlab/vulcan
WHdenovo	A cost-effective approach to diploid assembly for single samples and trios	https://github.com/shilpagarg/WHdenovo
Winnowmap	Long read/genome alignment software	https://github.com/marbl/Winnowmap
Winnowmap2	long-read mapping algorithm optimized for mapping ONT and PacBio reads to repetitive reference sequences	https://github.com/marbl/Winnowmap

### 7.3. Sequence assembly

It is crucial to produce annotated and complete genomes of microorganisms in order to gain a deeper understanding of their diversity and biology. The assembly method uses calculations such as sequence alignment and sequence merging to construct longer continuous sequences from short fragments of DNA. Specifically, sequence assembly compares the repeated regions between two pairs to splice the short sequenced fragment (read) obtained by base recognition into a longer continuous sequence (contig) and then splice the longer sequence into a longer skeleton (scaffolds) that allows blank sequence (gap). By eliminating skeleton errors and blank sequences, these skeletons are located on chromosomes to obtain high-quality whole genome sequences. This technology came into being because the nucleic acid molecules sequenced are usually much longer than existing DNA sequencing technologies. And this analysis aims to reconstruct the original appearance of the sequenced molecule from the DNA sequencing results of limited length. Nanopore sequencing has a longer read than the previous sequencing and can generate a large amount of data in one run.

Contrary to other sequencing platforms, nanopore sequencing does not exhibit bias in GC-rich regions and can cover repeat-rich sequences and structural variants that are not accessible to conventional sequencing methods. But it still cannot complete the sequencing of the entire genome in one sequencing, and there are many sequencing errors and genome duplications in the sequencing data. More error correction may be required to eliminate errors further and improve assembly accuracy, especially for the assembly methods without error correction steps. In addition, error correction can increase the local similarity between assembly and readings by mapping reads to assembly and changing assembly to improve the accuracy of draft assembly. Therefore, the top priority of sequence assembly is to deal with these problems reasonably and efficiently (El-Metwally et al., 2013).

With the continuous maturity of NST, there are increasingly open-source tools for assembly methods. A circular bacterial genome can be completed by CCBGpipe, according to this study (Liao et al., 2019). Creating contigs involves sampling reads and assembling them several times to create circular contigs that share a sequence. It is possible to assemble circular bacterial genomes using CCBGpipe automatically. In contrast to existing enhanced gap closure tools, which require multiple comparisons with high error rates, researchers in Xu et al. (2019) developed a software tool called TGS-GapCloser, which closes gaps without using any error correction, improving draft assembly N50 on average by 25 percent. With the combination of long reads and low-cost short reads, the POLCA assembly polishing tool can produce highly contiguous assemblies with low overall error rates. As an alternative, short reads can be incorporated during the assembly phase or used to polish the consensus that results from long reads (Zimin and Salzberg, 2020). By utilizing structural synteny between draft assemblies and reference sequences, the authors in Coombe et al. (2020) generate high-quality genome sequences. With NtJoin, a lightweight mapping approach is implemented based on an ordered minimizer sketch graph data structure. With NTJoin, highly contiguous assemblies can be generated more quickly and with less memory usage than existing reference-guided assemblers. According to Vaser and Šikić (2021), effective methods exist for improving *de novo* genome assembly from erroneous long-reads.

Raven, the tool presented in this paper, is one of the fastest options while consuming the lowest amount of memory. This paper (Quince et al., 2020) describes a new pipeline for identifying *de novo* strains when multiple metagenome samples from a given community are available. The coassembly graph is uniquely stored before the simplification of variants, which prevents ambiguities in the read mapping and utilizes more information about the cooccurrence of variants in reads than if variants were treated separately. In Fritz et al. (2021), several researchers describe Haploflow as a de Bruijn graph-based assembler for assembling viral genomes from mixed sequence samples. Compared to generic metagenomic assemblers and viral haplotype assemblers, Haploflow is both faster and more accurate.

In contrast to previous work, in Faure et al. (2021), the authors present GraphUnzip, a fast, memory-efficient, and accurate tool that generates high-quality gap-less supercontigs by connecting only sequences that overlap potential links. In Murigneux et al. (2021), the authors present an easy-access, integrated solution for attaining high-quality bacterial genomes by combining ONT with Illumina sequencing to create MicroPIPE, an end-to-end process for assembling bacterial genomes. However, the development of relevant bioinformatic tools, mainly quantitative analysis tools, is still insufficient. Therefore, the appropriate sequence assembly tool for nanopore sequencing should be selected based on the pathogenic microorganisms of specific diseases to achieve better results. We have summarized several popular tools currently available for assembling nanopore sequencing reads for reference purposes.

**Table 3:** 

Tool	Description	Availability
ABruijn	Find overlaps between reads without correcting	https://github.com/bioreps/ABruijn
ALLPATHS-LG	Uses hybrid and de Bruijn graphs	https://software.broadinstitute.org/allpathslg/blog
Bookend	End-guided transcriptome assembly	https://github.com/Gregor-Mendel-Institute/bookend
Canu	High-noise single-molecule sequencing	https://github.com/marbl/canu
CCBGpipe	The pipeline to complete circular bacterial genomes using a sampling strategy from a single MinION with barcoding	https://github.com/jade-nhri/CCBGpipe
CentroFlye	An algorithm for centromere assembly using long error-prone reads	https://github.com/seryrzu/centroFlye
Circlator	Circularizes genome assemblies	https://github.com/sangerpathogens/circlator
CulebrONT	A snakemake pipeline, able to launch multiple assembly tools in parallel	https://github.com/SouthGreenPlatform/culebrONT
DBG2OLC	Assembly of Large Genomes Using Long Erroneous Reads of the Third Generation Sequencing Technologies	https://github.com/yechengxi/DBG2OLC
DR2S	An R package designed to facilitate generating reliable, full-length phase-defined reference sequences for novel HLA and KIR alleles	https://github.com/DKMS-LSL/dr2s
ELBA	Distributed-Memory Parallel Contig Generation for *De Novo* Long-Read Genome Assembly	https://github.com/PASSIONLab/ELBA
Fast-SG	Alignment-free algorithm for hybrid assembly	https://github.com/adigenova/fast-sg
FinisherSC	A repeat-aware tool for upgrading *de novo* assembly using long reads	https://github.com/kakitone/finishingTool
Flye	Assembler, a combination of de Bruijn graph and OLC approach	https://github.com/fenderglass/Flye
GoldRush	A long read *de novo* genome assembler	https://github.com/bcgsc/goldrush
Haploflow	A strain-aware viral genome assembler for short read sequence data	https://github.com/hzi-bifo/Haploflow
HASLR	A hybrid tool for assembling genomes using long reads and short reads	https://github.com/vpc-ccg/haslr
HiCanu	An assembler of segmental duplications, satellites, and allelic variants from high-fidelity long reads	https://github.com/marbl/canu
Hinge	An OLC (Overlap-Layout-Consensus) assembler	https://github.com/HingeAssembler/HINGE
LazyB	A hybrid genome assembly	https://github.com/TGatter/LazyB
MaSuRCA	Combines the benefits of de Bruijn graph and OLC assembly approaches	https://github.com/alekseyzimin/masurca
MdBG	Minimizer-space de Bruijn graphs (mdBG) for whole-genome assembly	https://github.com/ekimb/rust-mdbg/
MicroPIPE	a pipeline for high-quality bacterial genome construction using ONT and Illumina sequencing	https://github.com/BeatsonLab-MicrobialGenomics/micropipe
MUFFIN	A pipeline for hybrid assembly and differential binning workflow for metagenomics, transcriptomics and pathway analysis	https://github.com/RVanDamme/MUFFIN
NextDenovo	A string graph-based *de novo* assembler for long reads	https://github.com/Nextomics/NextDenovo
NGSEP	Long-read *de novo* assembly	https://github.com/NGSEP/NGSEPcore
NpGraph	Streaming assembly for MinION data	https://github.com/hsnguyen/assembly
NPGREAT	NanoPore Guided REgional Assembly Tool	https://github.com/eleniadam/npgreat
NpScarf	Scaffolding and completing assemblies in real time	https://github.com/mdcao/npScarf
ntJoin	Scaffolding draft assemblies using reference assemblies and minimizer graphs	https://github.com/bcgsc/ntjoin
ONTrack	Rapid consensus assembly from MinION reads	https://github.com/MaestSi/ONTrack
OPERA-MS	A hybrid metagenomic assembler	https://github.com/CSB5/OPERA-MS
PECAT	Diploid assembly	https://github.com/lemene/PECAT
Peregrine-2021	A genome assembler designed for long-reads that have good enough accuracy	https://github.com/cschin/peregrine-2021
Phasebook	haplotype-aware *de novo* assembly of diploid genomes from long reads	https://github.com/phasebook/phasebook
Platanus-Allee	A *de novo* haplotype assembler	http://platanus.bio.titech.ac.jp/platanus2
PoreSeq	*De novo* sequence using the discretized ionic current data from an arbitrary number of independent nanopore reads of the same region of DNA	https://github.com/tszalay/poreseq
Purge Haplotigs	Allelic contig reassignment for third-gen diploid genome assemblies	https://bitbucket.org/mroachawri/purge_haplotigs
Ra	An OLC-based DNA assembler of long uncorrected reads (short for Rapid Assembler). Composed of Minimap2, Rala and Racon	https://github.com/rvaser/ra
RaGOO	Fast reference-guided scaffolding of genome assembly contigs	https://github.com/malonge/RaGOO
RagTag	Reference-guided genome assembly and correction, the successor of RaGOO	https://github.com/malonge/RagTag
Raven	A *de novo* genome assembler for long uncorrected reads	https://github.com/lbcb-sci/raven
RNA-Bloom2	Reference-free, long-read transcriptome assembly	https://github.com/bcgsc/RNA-Bloom
SDA	A Segmental Duplication Assembler for long reads	https://github.com/mvollger/SDA
Shasta	*De novo* assembly from primarily Oxford Nanopore reads and also from PacBio. The static executable is installation-free.	https://github.com/chanzuckerberg/shasta
StringTie2	A script assembly and quantification for RNA-Seq	https://github.com/mpertea/stringtie2
TGSGapFiller	A gap-closing software tool that uses error-prone long reads generated by third-generation-sequence techniques	https://github.com/BGI-Qingdao/TGSGapFiller
TULIP	Assembler for long reads	https://github.com/Generade-nl/TULIP
Wengan	A genome assembler	https://github.com/adigenova/wengan
Wtdbg2	A fuzzy de Bruijn graph approach to long noisy reads assembly	https://github.com/ruanjue/wtdbg2

### 7.4. SNP and variant detection

Each individual of a given species is unique at the genomic level. For example, the difference between us and others is approximately 3 million bp, or 0.1 percent. Therefore, reference genomes are often represented as common sequences from several people. However, individual features or illness susceptibility are caused by subtle changes in the DNA. SNPs, tiny insertions or deletions (indels), structural variation, such as big indels, and complicated rearrangements, such as translocation and inversion, are examples of genetic variation.

Structure variants (SVs) are a focus of many research fields, from cancer research to identifying SVs and encoding desired traits in crops. In most cases, SVs are up to megabases in size, so sequencing in short sections and reassembling them is impossible. Consequently, incomplete or incorrect assemblies may result, while PCR requirements may prevent SVs from arising in impossible-to-amplify regions. Read lengths are not limited with Oxford Nanopore: single reads frequently reach hundreds of kilobases, with the current record exceeding four megabytes. Therefore, even large SVs can often be sequenced end-to-end in single reads, allowing for precise characterization and often eliminating the necessity of assembly. In addition to identifying SVs throughout the genome without amplification, this method can identify repeat expansions and repetitive regions.

In this way, intact modified bases can be sequenced, allowing the identification of SVs and their epigenetic effects in a single experiment. In this regard, Oxford Nanopore Technology offers high scalability and is highly effective for studying SVs. For example, in Aljabr et al. (2020), researchers used Oxford nanopore long-read sequencing in conjunction with amplicon-based sequencing for rapid sequencing of MERS-CoV, providing data on consensus genomes, small mutations, and deletion mutants of MERS-CoV. Using this method, it is possible to identify insertions and deletions responsible for genotypic variations in coronavirus. Despite this, nanopore technology is not without its challenges. For example, the analysis of sequenced cancer genomes poses many challenges. In particular, mutations identified by multiple variant callers are often inconsistent, even though they use the same genome sequencing data.

Furthermore, read mapping and variant calling are required to identify somatic mutations, which are complicated processes. As a result, a simple method was developed for evaluating cancer genome sequencing data using K-mers sequences (Lee et al., 2020). This method validates mutations by comparing the frequency of mutations in k-mers between normal and tumor sequences that have been matched statistically.

Furthermore, in addition to the above challenge, as noisy long reads result in complex SV signatures, it is difficult to achieve both high throughput and excellent performance simultaneously. As such, the study (Jiang et al., 2020) proposes cuteSV, a sensitive, fast, and scalable method for detecting long-read signatures of SVs with high sensitivity and scalability over existing state-of-the-art tools, using a custom approach to collect signatures of various types of SVs. A further challenge is the high error rate of Oxford nanopores shown in the study (Feng et al., 2021); igda is used to compensate for the gap by detecting and phasing small single-nucleotide variants at frequencies as low as 0.2%. This method is significant in understanding cytogenetic heterogeneity within a species based on long-read metagenomic information. When applied to 4 × ONT WGS data, SV calling software often fails to detect pathogenic SV, particularly when the SV is composed of long deletions, end deletions, duplications, and unbalanced translocations. As shown in Leung et al. (2022), SENSV, a newly developed software for SV calling, is highly sensitive to all types of SV and has a breakpoint accuracy of typically 100 base pairs, both of which are concerning. Moreover, we have provided a list of some of the most commonly used variant calling tools below for your convenience.

**Table 4:** 

Tool	Description	Availability
AsmVar	A software for discovery, genotyping, and characterization of structural variants	https://github.com/bioinformatics-centre/AsmVar
Assemblytics	Software to identify structural variations based on contigs with uniquely aligned regions to a reference sequence	https://github.com/marianattestad/assemblytics
Clair	A program for rapid and accurate detection of small germline variants using single molecule sequencing data	https://github.com/HKU-BAL/Clair
Clairvoyante	a multi-task convolutional deep neural network for variant calling in Single Molecule Sequencing	https://github.com/aquaskyline/Clairvoyante
Davidcoffey-MinION	A pipeline for calling structural variants	https://github.com/davidcoffey/MinION
DeBreak	Deciphering the exact breakpoints of structural variations using long sequencing reads	https://github.com/Maggi-Chen/DeBreak
DeepVariant	Deep learning-based SNV detection that can be applied to long reads	https://github.com/google/deepvariant
Flopp	Long-Read Polyploid Haplotype Phasing by Uniform Tree Partitioning	https://github.com/bluenote-1577/flopp
FreeBayes	Haplotype-based variant detector	https://github.com/ekg/freebayes
Jasmine	Jointly Accurate Sv Merging with Intersample Network Edges	https://github.com/mkirsche/Jasmine
Last-Genotype	Identify substitutions and assign reads by genotype	https://github.com/mcfrith/last-genotype
LongPhase	ultra-fast chromosome-scale phasing algorithm for small and large variants	https://github.com/twolinin/LongPhase/
Longshot	A diploid SNV caller for error-prone reads	https://github.com/pjedge/longshot
LRcaller	A long-read structural variant caller	https://github.com/DecodeGenetics/LRcaller
Merfin	Improved variant filtering and polishing *via* k-mer validation	https://github.com/arangrhie/merfin
NanoCaller	A tool for detection of SNPs and indels in difficult-to-map regions from long-read sequencing by haplotype-aware deep neural networks	https://github.com/WGLab/NanoCaller
Nanopanel2	A somatic variant caller for nanopore panel sequencing data	A somatic variant caller for nanopore panel sequencing data
NanoSV	Identifies split- and gapped-aligned reads and clusters the reads according to the orientations and genomic positions of the read segments to define breakpoint-junctions of structural variations	https://github.com/mroosmalen/nanosv
NanoVar	A structural variant caller using low-depth Nanopore sequencing	A structural variant caller using low-depth Nanopore sequencing
NpInv	Detection of genomic inversions	https://github.com/haojingshao/npInv
Ococo	Capable of inferring variants in real-time, as read alignments are fed in	https://github.com/karel-brinda/ococo
RMETL	A realignment-based Mobile Element insertion detection Tool for Long read	https://github.com/tjiangHIT/rMETL
Smartie-Sv	calling SVs from Blasr contig level alignments	https://github.com/zeeev/smartie-sv
Sniffles	Detection of structural variants	https://github.com/fritzsedlazeck/Sniffles
Super-Minityper	An SV graph analysis on GPUs or in the cloud	https://github.com/NCBI-Codeathons/super-minityper
SVIM	Structural Variant Identification Method using Long Reads	https://github.com/eldariont/svim
SVision	Structural variant caller	https://github.com/xjtu-omics/SVision
SVJedi	Genotyping structural variations with long-read data	https://github.com/llecompte/SVJedi
SyRI	Synteny and Rearrangement Identifier	https://github.com/schneebergerlab/syri
TRiCoLOR	A tandem repeats profiler for long-read sequencing data	https://github.com/davidebolo1993/TRiCoLOR
VISOR	A haplotype-aware structural variants simulator for short, long and linked reads	https://github.com/davidebolo1993/VISOR
Vulcan	A long-read mapping and structural variant calling *via* dual-mode alignment	https://gitlab.com/treangenlab/vulcan

### 7.5. Signal analysis and visualizations

The signal processing of NST mainly concerns reducing noise and improving accuracy, and many new research activities have emerged in recent years.

Some researchers have optimized the Adaptive Band Event Alignment algorithm to run efficiently on heterogeneous CPU-GPU architectures (Gamaarachchi et al., 2020). A general nanopore method based on the combination of liposome signal amplification controlled by analyte and report molecule nanopore detection has been reported. This method greatly expands the application range of nanopores, and it is easy to change the sensitivity of nanopores from level μM to level fM (Tian et al., 2019). An analytical method for nanopores based on the Central Limit Theorem (CLT). The optimal voltage used in the detection is determined by the standard deviation of the blocking current and time constant under different voltage offsets. Compared with the traditional data analysis methods, the blocking signals processed by CLT result in a more concentrated distribution of blocking current and duration. It allows fitting Gauss to the duration histogram and avoids the influence of the box size on the time constant in duration analysis (Yan H. et al., 2021). CpelNano, a new statistical method, uses a hidden Markov model in which the true but unknown (“hidden”) measurement state is modeled through an Ising probability distribution that is consistent with measurement means and pair correlations, where as nanopore current signals are consistent with the observed state (Abante et al., 2021). Some researchers combined mixed chain reaction (HCR) with nanopore detection to transform the existence of small DNA targets into characteristic nanopore signals of long-nicked DNA polymers. The amplification of the nanopore signal obtained through HCR not only overcomes the functional limitations of solid nanopores but also significantly improves the selectivity and signal-to-noise ratio, thus allowing the detection of ctDNA at the detection limit of 2.8 fM (S/N = 3) and single-base resolution (Sun et al., 2020). Other researchers have proposed a new robust method, which can accurately classify the original nanopore signal data by converting the current intensity into images or pixel arrays and then using the depth learning algorithm to classify. The development of the first experimental protocol for direct RNA sequencing library barcode multiplexing proves the power of this strategy (Smith et al., 2020). Scientists have proposed SquiggleNet, which is the first deep learning model that can directly classify nanopores from their electrical signals. SquiggleNet runs faster than DNA through pores, allowing real-time sorting and reading jets. SquiggleNet distinguishes human and bacterial DNA with more than 90% accuracy, extends it to bacterial species not found in human respiratory system metagenomic samples, and accurately classifies sequences containing human long interpenetrating repeats (Bao et al., 2021). A deep learning method for removing ion current noise in resistance pulse sensors is presented. The electric displacement of a single nanoparticle in the nano-corrugated nanopore was detected. The noise is reduced by the convolutional auto-coding neural network, which is designed to optimize iterative comparison and minimize the difference between a pair of waveforms through gradient descent. Denoising in high-dimensional feature space has proved to be able to detect waveform signals derived from ripples. These signals cannot be identified in the original curve or frequency domain under the given noise baseline after digital processing, so the electrokinetic analysis of rapidly moving single nanoparticles and double nanoparticles can be tracked *in situ*. The ability of label-free learning to remove noise without affecting time resolution may be helpful in solid-state nanopore sensing of protein structure and polynucleotide sequence (Tsutsui et al., 2021).

New research also emerges in the field of nanopore sequencing data visualization. BulkVis is a tool that can load batch Fast5 files, cover MinKNOW (software that controls the ONT sequencer) classification on signal tracking, and display the mapping to reference. The user can navigate to a channel and time or jump from reading to a specific location in the case of a given FASTQ header. BulkVis can export a region as a read compatible with the Nanopole basic caller (Payne et al., 2019). Sequoia, a visual analysis tool, allows users to explore nanopore sequences interactively. Sequoia combines the Python-based back-end with the multi-view visualization interface, enabling users to import original nanopore sequencing data in Fast5 format, cluster sequences based on current similarity, and go deep into the signal to identify the attributes of interest (Koonchanok et al., 2021). A portion of the associated tools are as follows:

**Table 5:** 

Tool	Description	Availability
BaseLess	lightweight detection of sequences in raw nanopore data	https://github.com/cvdelannoy/baseLess
f5c	An optimized re-implementation of the index, call-methylation, and eventalign modules in Nanopolish	https://github.com/hasindu2008/f5c
CLT	An analytical method for nanopores based on the Central Limit Theorem (CLT)	https://github.com/xiangyan585/CLT.git
PyPore	Tools to analyze Nanopore signal data	https://sourceforge.net/projects/pb-jelly/
Sequoia	A web interface tool for visualizing similarities of nanopore sequencing data	https://github.com/jmschrei/PyPore
Sigtk	A simple toolkit performing various operations on nanopore raw signal data	https://github.com/hasindu2008/sigtk/
SquiggleKit	A toolkit for accessing and manipulating nanopore signal data	https://github.com/Psy-Fer/SquiggleKit
SquiggleNet	1D ResNet based model to classify Oxford Nanopore raw electrical signals as target or non-target for Read-Until sequence enrichment or depletion	https://github.com/welch-lab/SquiggleNet
Betaduck	For tidying the PromethION outputs and visualizing the data	https://github.com/alexiswl/betaduck
BulkVis	An app to visualize raw squiggle data from Oxford Nanopore Technologies (ONT) bulk files	https://github.com/LooseLab/bulkVis
D-GENIES	An online tool designed to compare two genomes. It supports large genome and you can interact with the dot plot to improve the visualisation	http://dgenies.toulouse.inra.fr/
Methylartist	Tools for parsing and plotting methylation patterns from nanopore data	https://github.com/adamewing/methylartist
Minidot	Creates dotplots from minimap2 results	https://github.com/thackl/minidot
Modbamtools	set of tools to manipulate and visualize data from base modification bam files	https://github.com/rrazaghi/modbamtools
NanoMethViz	A toolkit for visualizing methylation data from Oxford Nanopore sequencing	https://github.com/Shians/NanoMethViz
NanoPack	A set of tools developed for visualizing and processing long-read sequencing data from Oxford Nanopore Technologies and Pacific Biosciences	https://github.com/wdecoster/nanopack
NanoPlot	Plotting reads and alignments	https://github.com/wdecoster/NanoPlot
Plotnanopolish	Scripts for plotting nanopolish methylation data	https://github.com/gilfunk/plotnanopolish
Plotsr	A tool for visualization of synteny and structural rearrangements between multiple genomes	https://github.com/schneebergerlab/plotsr
PycoQC	pycoQC computes metrics and generates Interactive QC plots from the sequencing summary report generated by Oxford Nanopore technologies basecaller (Albacore/Guppy)	https://github.com/a-slide/pycoQC
R_poRe	Extraction and visualization of nanopore sequencing data	https://sourceforge.net/projects/rpore
Streamformatics	NOT MAINTAINED:: Real-time species-typing visualization for nanopore data.	https://github.com/mbhall88/streamformatics
Swan	A python library to visualize and analyze long-read transcriptomes	https://github.com/mortazavilab/swan_vis

### 7.6. Adaptive sampling

Adaptive sampling, or selective sequencing, allows for rapid, flexible, adaptive sequencing of numerous regions of large genomes or specific subsets of multiple genomes because of the ability to reject individual molecules while they are being sequenced (Loose et al., 2016). By reducing the time and cost of both sequencing and sample preparation, these methods enable researchers to focus long-read sequencing on specific regions to address biological concerns.

Several relevant studies have already been conducted in this field. Unlike previous pattern matching methods, RUBRIC operates in sequence space, allowing for real-time selective sequencing for Oxford MinION. Furthermore, RUBRIC’s pre-screening feature reduces computational requirements by allowing only informative and timely reads to be admitted during the decision process, thereby enabling real-time base finding, alignment, and selection of MinION reads without needing a dedicated high-performance computing environment (Edwards et al., 2019). In addition, researchers in Thomsen et al. (2019) have created a rationally designed DNA nanopore with the largest channel lumen to date, which has been combined with a programmable trigger, significantly expanding the functionality of nanopores. They demonstrate a size-selective, modular, and responsive gating mechanism for molecules between compartments separated by lipid bilayers based on direct observation and detailed analysis of the insertion and translocation kinetics of individual DNA nanopores on liposomes. Another method called UNCALLED can quickly match flow nanopore current signals with reference sequences about flows. With an FM-index as a basis, UNCALLED considers the possibilities of the signal representing one of the K-mers and prunes the candidates accordingly based on the probabilistic analysis (Kovaka et al., 2021). Meanwhile, using the Oxford Nanopore Technology, T-LRS was able to resolve mutations more precisely using adaptive sampling, and it has proven to be an efficient and cost-effective way to analyze genes and areas of high priority, which leads to better quality data (Miller et al., 2021). Also, researchers in Wanner et al. (2021) employed nanopore adaptive sampling to sequence the L. rosalia mitogenome from feces, a non-invasive sample. Compared to sequencing without enrichment, adaptive sampling resulted in a two-fold increase in both the fraction of host-derived and mitochondria-derived sequences. A synthetic mock metagenomic community and a complex real sample were enriched using ONT’s adaptive sampling software. Targeted enrichment on low-abundance species significantly reduced the time required to assemble high-accuracy, single-contigs compared to non-targeted sequencing. Their findings show that repeated ejections of molecules from pores have less impact on pore stability than previously reported, and nanopore-based metagenomic studies will benefit from adaptive sampling (Martin et al., 2022). A novel approach called ReadBouncer to nanopore adaptive sampling based on interleaved bloom filters, and fast CPU and GPU base calling is presented here. By enhancing the sensitivity and specificity of reading classification, ReadBouncer enhances the potential enrichment of the low abundance sequences, surpassing the capabilities of existing tools available in the field. While the software runs on commodity hardware without GPUs, it is robust enough to remove even reads belonging to large reference sequences. This makes it accessible to researchers working in the field (Ulrich et al., 2022). The utility of RPNAS for enriching viral genomes from clinical samples has been highlighted by some researchers. By using adaptive sequencing, specific genome data could be obtained, and the recovery time could be shortened, which could be applied to pathogen detection and surveillance of infectious diseases at the point-of-care with high sensitivity and short turnaround times (Lin et al., 2022). For your convenience, we have included a list of some of the most commonly used adaptive sampling tools below:

**Table 6:** 

Tool	Availability
ReadBouncer	https://github.com/JensUweUlrich/ReadBouncer
ReadFish	https://github.com/looselab/readfish
Read-Until	https://github.com/mattloose/RUscripts
RUBRIC	https://github.com/harrisonedwards/RUBRIC
T-LRS	https://github.com/danrdanny/targetedLongReadSequencing
UNCALLED	https://github.com/skovaka/ UNCALLED

### 7.7. Epigenetic modification detection

Epigenetic modification refers to the heritable changes in gene expression caused by environmental factors without changing the DNA sequence (Kader and Ghai, 2015). Epigenetic modifications mainly include DNA methylation, DNA-protein interactions, chromatin accessibility, histone modifications, and more (Alizadeh et al., 2017). From DNA to histones to RNA, these chemical modifications that occur at different dimensions interact and ultimately affect synapses, memory, and neural pathways. In addition, they affect changes in proteins related to neural signaling. In order to advance biomedical research, it is necessary to have a thorough understanding of epigenetic mechanisms, their interactions, and their alterations in health and disease.

By analyzing DNA methylation, researchers are able to gain valuable insights into gene regulation and identify potential biomarkers. Several diseases, including cancer, obesity, and addiction, have been associated with aberrant DNA methylation. Initially, DNA methylation studies focused on identifying the positions of methylation of examined genes and determining the amount of 5-methylcytosine present in DNA. 5-methyl cytosine and nonmethylated bases are distinguished by bisulfite treatment, which is a widely used and effective method (Zhang et al., 2015). The use of bisulfite for the detection of 5-methyl cytosine, although widely used, does have certain limitations, including incomplete conversion, false-positive results, and difficulty in using (Laird et al., 2016; Gholizadeh et al., 2017). Since nanopore sequencing does not require PCR amplification, base decorations can be detected simultaneously in the nucleotide sequence without additional sample preparation. Furthermore, nanopore sequencing is free of GC bias, making it easier to obtain consistent sequencing depths and to identify regions of the genome that cannot be identified by traditional sequencing methods and methylation-calling techniques. Compared to bisulphite data, nanopore methylation analysis yields lower bias, better mapping rates, superior reproducibility, and faster analysis. Consequently, more analytical tools are being developed to detect DNA methylation in nanopore sequencing data. When using nanopore data for DNA methylation detection, a methodological challenge arises, namely detecting DNA modifications in non-singleton CpG sites. The difference between singletons and non-singletons is the number of CpG sites per 10-basepair region. The methylation status of CpGs within a 10-bp region is assumed to be the same, so identifying changes in CpGs located near one another is challenging. Although several methods (as listed in the table) have been developed to detect DNA-methylation in singletons, it remains difficult to detect DNA-methylation in non-singletons (Simpson et al., 2017; Ni et al., 2019). Meanwhile, the level of DNA methylation varies depending on the genomic context and does not follow a linear distribution (Li and Zhang, 2014; Greenberg and Bourc’his, 2019), and different genomic regions may yield different methylation results. To this end, a comprehensive survey provides a systematic comparison of next-generation methylation calling tools and provides guidelines for their development (Liu Y. et al., 2021). As part of our contribution, we also provide relevant available tools to assist in detecting epigenetic modifications.

**Table 7:** 

Tool	Description	Availability
CfNano	Deconvolute cell types from cell-free DNA methylation	https://github.com/methylgrammarlab/cfdna-ont
CHEUI	Identification of m6A and m5C RNA modifications at single-molecule resolution from Nanopore sequencing	https://github.com/comprna/CHEUI-public
DeepMod	Detection of base modifications in ONT data	https://github.com/WGLab/DeepMod
DeepMP	DNA base modifications on Nanopore sequencing data	https://github.com/pepebonet/DeepMP
DeepSignal-Plant	Detecting DNA methylation state from Oxford Nanopore sequencing reads of plants	https://github.com/PengNi/deepsignal-plant
DENA	training an authentic neural network model using Nanopore sequencing data of Arabidopsis transcripts for detection and quantification of N6-methyladenosine on RNA	https://github.com/weir12/DENA
Differr	Detecting modifications from Nanopore direct RNA-sequencing errors using a low modification control	https://github.com/bartongroup/differr_nanopore_DRS
DNAModAnnot	R toolbox for DNA modification filtering and annotation	https://github.com/AlexisHardy/DNAModAnnot
DRUMMER	Identify RNA modifications at the nucleotide-level resolution on distinct transcript isoforms through the comparative analysis of basecall errors in Nanopore direct RNA sequencing (DRS) datasets.	https://github.com/DepledgeLab/DRUMMER
EpiNano	Detection of RNA modifications from Oxford Nanopore direct RNA sequencing reads	https://github.com/enovoa/EpiNano
F5c	A faster implementation of Nanopolish call-methylation in C	https://github.com/hasindu2008/f5c
Fast5Mod	A set of two programs for converting Guppy’s modified base Fast5 output into an aligned or unaligned BAM formatted file, and for aggregating modified base calls	https://github.com/nanoporetech/fast5mod
Haplotyped-Methylome	Using long-read sequencing to detect imprinted DNA methylation	https://github.com/scottgigante/haplotyped-methylome
MCaller	A binary classifier to improve bacterial base modification N6-methyladenine (m6a) classification by marking adenines as methylated or unmethylated based on differences between measured and expected current values as each adenine travels through the nanopore.	https://github.com/al-mcintyre/mCaller
Megalodon	Anchors raw nanopore signal to reference to call SNP and modified bases	https://github.com/nanoporetech/megalodon
MeMoRe	Methylation Motif Refiner. This is a tool designed to validate and, if necessary, refine methylation motifs detected from Bacteria and Archaea with SMRT and ONT sequencing.	https://github.com/fanglab/MeMoRe
METEORE	MEthylation deTEction with nanopORE sequencing. Automatic DNA methylation detection from nanopore tools and their consensus model	https://github.com/comprna/METEORE
MethBERT	Tool for nanopore 5mC/6 mA detection	https://github.com/yaozhong/methBERT
Methylartist	Tools for parsing and plotting methylation patterns from nanopore data	https://github.com/adamewing/methylartist
Nanocompore	A tool that identifies differences in ONT nanopore sequencing raw signal corresponding to RNA modifications by comparing 2 samples	https://github.com/tleonardi/nanocompore
Remora	Methylation/modified base calling separated from basecalling	https://github.com/nanoporetech/remora
SignalAlign	This paper presents one of the first methods able to detect methylation changes directly from Oxford Nanopore long-read sequencing.	https://github.com/ArtRand/signalAlign
Tombo	A suite of tools primarily for the identification of modified nucleotides	https://github.com/nanoporetech/tombo

### 7.8. Compression and file formats

In spite of the fact that nanopore sequencing is reshaping the genomic landscape, enormous volumes of genomic sequencing data have been generated in recent decades that require a great deal of storage space. Due to the difficulty in storing and transferring such data, bottlenecks have occurred in genome sequencing analyses.

In this regard, compression has become an essential aspect of storing, transmitting, and analyzing data. Currently, compression methods that focus on short-read long sequencing are no longer applicable to genomics due to the dominance of long-read long sequencing. This has prompted researchers to study long-read long compression techniques in greater depth. To reduce the size of raw FASTQ raw sequencing data, several compression techniques have been proposed, including Picopore, ENANO, RENANO, NanoSpring, FastqCLS, and CoLoRd (Gigante, 2017; Dufort y Álvarez et al., 2020, 2021; Meng et al., 2021; Kokot et al., 2022; Lee and Song, 2022). As an example, Picopore (Gigante, 2017) provides a software suite that contains three compression methods: raw, lossless and deep lossless compression. All of these methods are capable of being performed simultaneously with sequencing, thereby reducing the need for immediate and long-term storage. The ENANO algorithm (Dufort y Álvarez et al., 2020), which focuses on mass fraction compression and provides both maximum and fast compression modes, trades off between the efficiency and speed of the compression process. In comparison to ENANO, RENANO (Dufort y Álvarez et al., 2021) achieves significantly improved compression of read sequences but is limited to aligned data with a usable reference. In contrast, NanoSpring (Meng et al., 2021) is a reference-free tool that relies on approximate assembly methods in order to achieve compression gains, but it requires more time and memory to accomplish compression gains. The FastqCLS (Lee and Song, 2022) compression algorithm uses read reordering to compress long reads of long sequencing data without sacrificing information and performs well in terms of compression ratio. CoLoRd (Kokot et al., 2022) uses data redundancy in overlapping reads to reduce the size of nanopore sequencing data by an order of magnitude without compromising downstream analysis accuracy. Furthermore, the paper (Gamaarachchi et al., 2022) argues that nanopore sequencing relies upon the Fast5 file format for efficient parallel analysis in order to address large data volumes and computational bottlenecks. In addition, it introduces SLOW5, an alternative format for efficiently parallelizing nanopore data and accelerating its analysis. As an example, on a typical high-performance computer, DNA methylation analysis of the human genome was reduced from over 2 weeks to roughly 10.5 h. Below is a table that provides access to the tools mentioned above.

**Table 8:** 

Tool	Availability
CoLoRd	https://github.com/refresh-bio/CoLoRd
ENANO	https://github.com/guilledufort/EnanoFASTQ
FastqCLS	https://github.com/krlucete/FastqCLS
NanoSpring	https://github.com/qm2/NanoSpring
Picopore	https://github.com/scottgigante/picopore
RENANO	https://github.com/guilledufort/RENANO
SLOW5	https://github.com/hasindu2008/slow5tools

### 7.9. Solid-state nanopore sequencing data analysis

While protein nanopores are limited by their short lifespan, solid-state nanopores mainly fabricated with SiNx, SiO_2_ or MoS_2_ have attracted plenty of research activities during the past decade (Goto et al., 2019). However, direct sequencing by solid-state nanopores is still challenging, mainly due to the ultrafast speed of molecule translocation and significant noises embedded in raw signals (Fragasso et al., 2020). As a consequence, the fundamental step toward solid-state nanopore sequencing data analysis is event detection, which aims to precisely locate the time range of molecule translocation and to determine different levels of translocation events driven by complex conformations such as DNA knots (Beamish et al., 2020; Wen et al., 2021).

Several automated tools have been proposed to perform event detection by considering the mean value and/or standard deviation of the current values, such as Transalyzer, EasyNanopore (Tu et al., 2021), and EventPro (Bandara et al., 2021). Recently, we have also developed two event detection tools focusing on the local range close to the peak current value in each segmented slice: the former uses a straight-forward statistical approach to select the real events from a considerable amount of slices (Sun et al., 2022), while the latter takes into account the ratio of the diameters of the molecule relative to that of the nanopore. In recent years, machine learning-based methods have also been proposed to perform event detection tasks, and a transformer-based method has been developed (Dematties et al., 2022), as well as a bi-path method to detect the events from low signal-to-noise current traces (Dematties et al., 2021), showing impressive performances.

Following event detection, several researchers have proceeded to employ machine learning methods to extract the event features and classify events driven by different molecules. A deep learning method to distinguish single nucleotides was developed (Díaz Carral et al., 2021); Another method was developed to identify different coronaviruses with high accuracy by combining solid-state nanopore structure and machine learning model (Taniguchi et al., 2021). This innovative sequencing platform has prompted the launch of a new commercial company, Aipore Inc. In a third study, researchers proved to succeed in using image recognition techniques to classify the events driven by four synthetic glycosaminoglycans (Xia et al., 2021). These pioneering studies have demonstrated that, in the future, artificial intelligence and machine learning techniques will play an increasingly important role in the development of solid-state nanopore sequencing.

### 7.10. Portable nanopore analytics

Following the miniaturization of integrated circuitry and other computer hardware over the past several decades, nanopore sequencing taking the shape of smaller size, portable or even hand-held form factors, has emerged on the horizon. This tendency launched a new era that laptop computers, tablets, and even smartphones and portable Android devices could provide computing resources to support nanopore sequencing (Palatnick et al., 2020). Since 5G technology boasts a secure and high data transmission rate, the research teams from Southeast University and China Mobile have embarked on a joint venture to combine nanopore sequencing with an integrated 5G network-cloud computing framework to perform online nanopore sequencing and data analytics away from the Lab and computing facilities.

## 8. Conclusion

The single molecule detection technology based on NST has been widely used for the analysis of a variety of biological samples and will be applied to more aspects of analysis in the future. As a promising next-generation sequencing technology, NST is expected to become a marker-free, fast and low-cost DNA sequencing technology, which will bring the world the hope of sequencing anything, anytime and anywhere. By way of nanopore-based technology to detect DNA sequences, researchers have been able to identify and analyze various types of analytes in animals, plants, bacteria, viruses (especially COVID-19 detection), etc., while requiring small sample preparation. Long read length (10000–50,000 bases), does not require amplification and modification (read the sequence directly, easy to operate), can be used in various environments, and has achieved good results. In biophysics and nano-biotechnology, biological and solid-state nanopores are effective single-molecule sensors. The use of solid-state nanopores in conjunction with other single-molecule techniques allows for a multi-modal approach to identifying tiny features of individual DNA molecules. Although nanopore sequencing has good operability and broad application prospects, some significant difficulties remain to be overcome. Among these is the demand for ultra-precise, high-speed DNA detection beyond the spatial and temporal resolutions of existing optical and electrical technologies, which is a key restriction of nanopore-based DNA sequencing at the single-molecule level. As a result, single-base recognition and DNA velocity reduction remain the primary problems. Nanopore technology will significantly impact DNA sequencing as well as the future of personal health and illness diagnosis. Integrating the advantages of nanopore experiment strategy, micromachining technology, circuit design, and the development of improved algorithms will hasten the advancement of nanopore technology toward inexpensive and tailored DNA detection, particularly DNA sequencing.

Currently, the high error rate is still the key to hindering its application. At present, the single-base accuracy of the MinION sequencer is about 85% (Cretu Stancu et al., 2017), and the accuracy of the revised consensus sequence is about 97% (Jain et al., 2018). For organisms with large genomes and relatively low mutation rates, such as animals and plants, increasing the sequencing depth can achieve a certain error correction effect, and the impact on the research results is not particularly large. However, clinical testing usually requires very strict data accuracy. Moreover, the virus genome is small, and the mutation rate is high, so the impact of the error rate cannot be ignored. Therefore, improving the accuracy of the nanopore sequencing platform so that it can better serve scientific researchers is still the direction of our efforts. Single-molecule NST needs to develop in the direction of higher throughput, higher accuracy, and a higher degree of automation.

Simultaneously, researchers and nanopore sequencing researchers need to work on enhancing and integrating current technologies and circumstances, as well as developing new technological methods and solutions for the study of biological challenges. We should consider our own research goals and needs, take advantage of existing platforms, learn from each other, and develop appropriate sequencing plans to achieve new scientific discoveries. Furthermore, the performance of sequencing technology should be constantly improved, as should the scope of application of sequencing technology based on actual and new experimental demands.

## Author contributions

PC, ZS, and JW: data curation, model creation, and paper draft. XL: design of the methodology. YB: formal analysis. JC, AL, and FQ: sample collection. YC, CY, JS, and JZ: manuscript review. L-QX and JL: overall project design, conceptualization, investigation, supervision, and revision of the paper. All authors contributed to the article and approved the submitted version.

## Funding

The academic authors acknowledge the financial support of the National Natural Science Foundation of China (under grant number 32270607). And all authors are grateful to China Mobile (Chengdu) Industrial Research Institute for funding the research (grant numbers 8531000015 and 8531000019). The author also thanks for supporting the Space Medical Experiment Project of China Manned Space Program (HYZHXM01004).

## Conflict of interest

The authors declare that the research was conducted in the absence of any commercial or financial relationships that could be construed as a potential conflict of interest.

## Publisher’s note

All claims expressed in this article are solely those of the authors and do not necessarily represent those of their affiliated organizations, or those of the publisher, the editors and the reviewers. Any product that may be evaluated in this article, or claim that may be made by its manufacturer, is not guaranteed or endorsed by the publisher.
